# Connecting signaling and metabolic pathways in EGF receptor-mediated oncogenesis of glioblastoma

**DOI:** 10.1371/journal.pcbi.1007090

**Published:** 2019-08-06

**Authors:** Arup K. Bag, Sapan Mandloi, Saulius Jarmalavicius, Susmita Mondal, Krishna Kumar, Chhabinath Mandal, Peter Walden, Saikat Chakrabarti, Chitra Mandal

**Affiliations:** 1 Cancer Biology and Inflammatory Disorder Division, Indian Institute of Chemical Biology, Kolkata, India; 2 Structural Biology and Bioinformatics Division, Indian Institute of Chemical Biology, Kolkata, India; 3 Department of Dermatology, Venerology and Allergology, Charité– Universitätsmedizin Berlin corporate member of Freie Universität Berlin, Humboldt-Universität zu Berlin, and Berlin Institute of Health, Berlin, Germany; 4 National Institute of Pharmaceutical Education and Research, Kolkata, India; German Cancer Research Center (DKFZ), GERMANY

## Abstract

As malignant transformation requires synchronization of growth-driving signaling (S) and metabolic (M) pathways, defining cancer-specific S-M interconnected networks (SMINs) could lead to better understanding of oncogenic processes. In a systems-biology approach, we developed a mathematical model for SMINs in mutated EGF receptor (EGFRvIII) compared to wild-type EGF receptor (EGFRwt) expressing glioblastoma multiforme (GBM). Starting with experimentally validated human protein-protein interactome data for S-M pathways, and incorporating proteomic data for EGFRvIII and EGFRwt GBM cells and patient transcriptomic data, we designed a dynamic model for EGFR-driven GBM-specific information flow. Key nodes and paths identified by *in silico* perturbation were validated experimentally when inhibition of signaling pathway proteins altered expression of metabolic proteins as predicted by the model. This demonstrated capacity of the model to identify unknown connections between signaling and metabolic pathways, explain the robustness of oncogenic SMINs, predict drug escape, and assist identification of drug targets and the development of combination therapies.

## Author summary

Complex and highly dynamic interconnected networks allow cancer to take different routes and circumvent chemotherapy. Therefore, understanding these context-specific networks and their dynamics of molecular interactions driven by different oncogenic signaling and metabolic pathways is very much needed to predict drug targets and the effect of therapeutics. We incorporated high-throughput transcriptome and proteome data into mathematical models to deduce properties of cancer cells through systems biology approach. Here we report the development, testing and validation of an integrated systems biology model of information flow between signaling and metabolic pathways to understand the regulation of the interconnection between them in cancer. Our model efficiently identified unique connections and key nodes important in signaling-metabolic information flow. We predicted some potential novel targets before performing actual drug tests. We have successfully applied this model to identify the interconnections altered in the constitutive signaling of the mutated EGFR by comparing EGF-dependent and wild-type EGFR signaling in glioblastoma multiforme.

## Introduction

Diseases like cancer involve a large range of components that interact via complex and highly dynamic networks [1–3], and are interconnected with biochemical pathways [4–7]. These multipath interconnections may allow cancer and other diseases to take alternate routes and bypass the effects of therapeutic interventions. Traditional approaches to biological studies which focus on single molecules or pathways may not be able to capture and understand these complex networks of molecular interactions. To predict alternative or escape routes around blockades and to develop effective therapies [8], sophisticated mathematical and computational models are required [9–10].

Transforming traditional drug discovery approaches toward smarter therapeutic strategies, the field of systems biology is emerging [1, 9, 11–20]. Systems approach generally involve large-scale data collections, most often from high-throughput transcriptome or proteome analyses, incorporation of the data into mathematical models to deduce systems properties, model building and finally computational and/or experimental validation of model-derived hypotheses. Systems biology approaches may predict combination therapies for cancers driven by different oncogenic signaling and metabolic pathways.

Signaling and metabolic networks were studied using separate model systems [15, 21–26]. Mathematical models for signaling pathways had been based on logic models [27–30], kinetic models [31–33], decision tree [34], and other differential equation-based models [35]. Computational models of molecular signaling [36–41] have the potential to improve drug discovery and development [32,42–44]. Analyses of knockdown experiments [45] using mass spectrometry [46] and transcriptomics [47–49] are progressively refined and tuned towards specific physiological situations. While these studies have helped considerably to extend our understanding of tumor biology, they are still restricted to signaling pathways and do not integrate the metabolic pathways, which in some initial studies have been subjected to separate systems biology analysis.

Predicting the effects of multiple targeted drugs [8, 50] with modeling the information flow from new molecular interactions within pathways is challenging [51–55]. Here we report the development, test and validation of an integrated model for signaling and metabolic pathways in cancer using glioblastoma multiforme (GBM) as an example [47, 56–59]. GBM is the most prevalent and most aggressive brain tumor. In the majority of cases, tumor development is dependent on signaling via the epidermal growth factor receptor (EGFR) and requires EGF in lower-grade forms or is EGF-independent in the more aggressive forms. In most cases, the expression of EGFR is up-regulated, often related to the amplification of the EGFR gene. More than fifty per cent of EGFR-amplified GBM cases have in-frame deletions of exons 2–7 that code for the extracellular ligand-binding domain (EGFRvIII mutation) resulting in EGF-independent constitutive signaling and more aggressive tumor growth, higher invasiveness, increased resistance to treatment, and poor prognosis [60–62].

In this study, we choose the cell line U87MG expressing the EGF-dependent EGFRwt and its derivative U87MGvIII expressing the EGF-independent EGFRvIII-mutant, as models of low and high-grade GBM, respectively. Except for the EGFR mutation, the two cell lines have the same genotype but differ in growth behavior suggesting different metabolic requirements, and are experimental examples for the regulation of the interconnection of signaling and metabolic pathways, which is considered among the basic characteristics of cancer [63–64].

**Fig 1:**
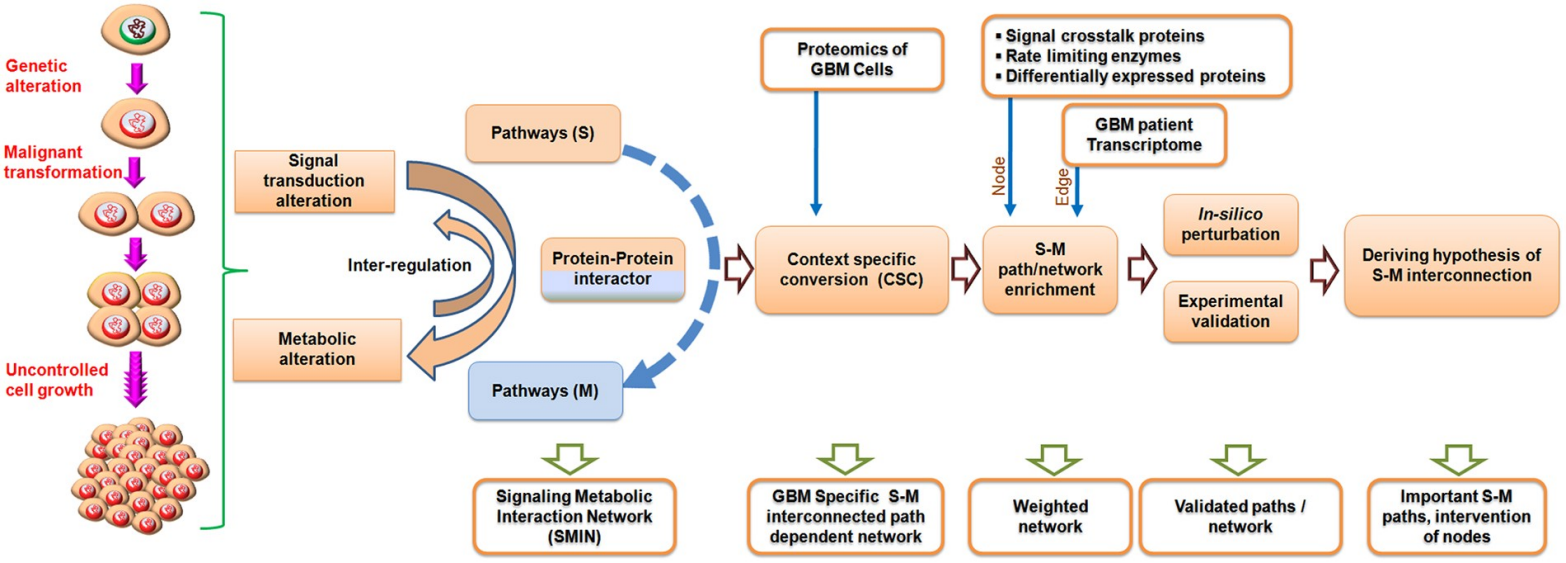
A schematic representation of the steps to establish the context-dependent signaling-metabolic interconnection model.

We implemented a probabilistic approach based on the Hidden Markov Model (HMM) utilizing the information of experimentally established protein-protein interactions (PPIs) [65–66] to extract novel paths and interconnections between signaling pathway proteins (S) and metabolic pathway proteins (M). To cope with the limitations of PPI identification, for example high error rates of the detection methods [67], incomplete data sets, ignorance of the physiological conditions in the cell or tissue compartments [68], technical problems and study biases [69], we collected information from curated sources (http://string-db.org) with high experimental score cut-off, which reduces false positive rates, and used transcriptome data from clinical samples to build more reliable and GBM context-specific PPI networks. To bridge the gap between transcriptome data and cell-biological processes, we incorporated proteome data from the GBM cell lines complemented with transcriptome data to solve the missing data problem caused by the failure of proteomics to capture all proteins in the cells due to sensitivity and reproducibility issues. Including experimental data, our dynamic model can make use of multiple weighted network properties to add biologically relevance and can extract novel paths of information propagation in networks. The results of the model were tested in rigorous *in-silico* perturbation experiments and experimentally validated in cell culture systems. Fig 1 depicts the overall strategy implemented with this study.

## Results

### Construction of a signaling to metabolic pathway interconnection network

**Fig 2:**
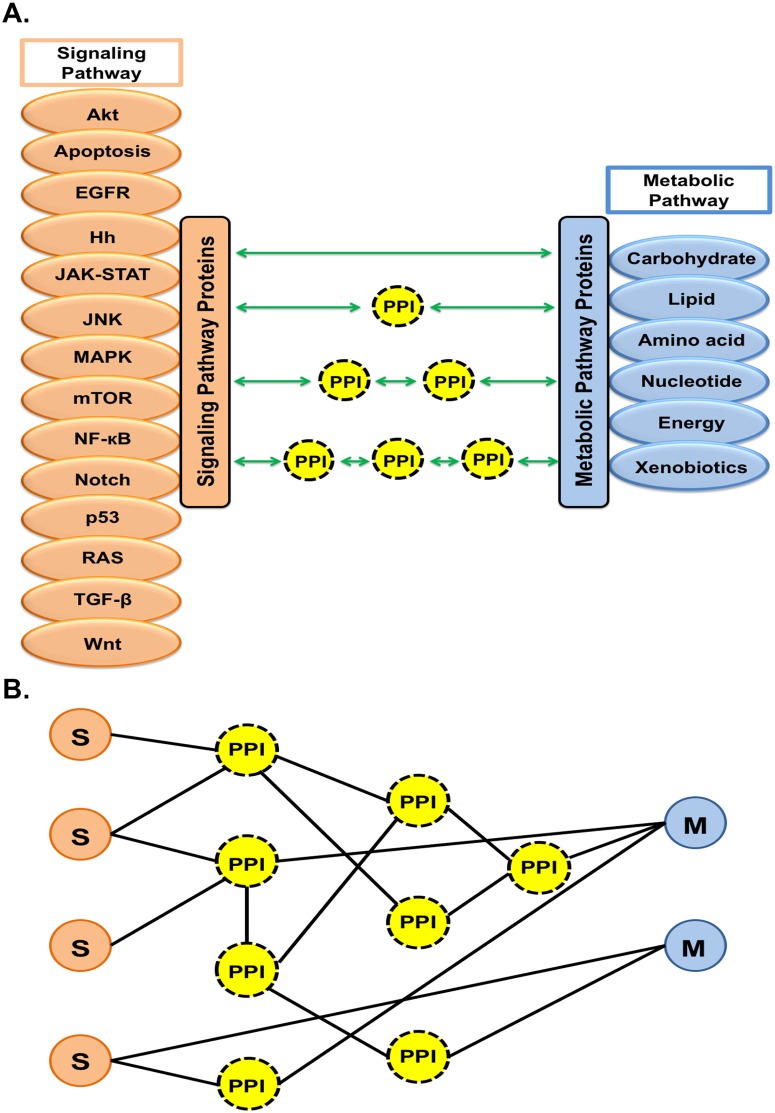
Establishment of signaling-metabolic pathway protein (S-M) interconnections for construction of SMIN. **(A**) Fourteen signaling (left) and six groups of metabolic pathways (right; grouping of 81 individual metabolic pathways) were selected from the KEGG database to develop the S-M interconnected network (SMIN) through PPI. Experimentally validated data from the STRING protein-interaction database were used to construct an initial HPPIN. The levels of interaction were restricted at the interactors of interactors to create the network resulting in four possible linking paths through which selected signaling and metabolic pathway proteins could be connected directly or through one, two and three PPI. (**B)** Conversion of interconnecting paths into network representation.

As a starting model, we constructed an integrated network where signaling (S) and metabolic (M) pathway proteins were connected through protein-protein interactors (PPIs). The signaling pathways were the apoptosis, Akt, EGFR, hedgehog (Hh), JAK-STAT, JNK, MAPK, mTOR, NF-kappa B (NF-κB), Notch, p53, Ras, TGF-β, and Wnt pathways. On the metabolic site, there were 81 pathways grouped into the six categories carbohydrate, lipid, amino acid, nucleotide, energy and xenobiotic metabolism (Fig 2).

To build an initial human protein-protein interactome network (HPPIN), the total of all human protein-protein interactions was extracted from the protein-interaction database STRING [70] for experimentally validated interactions including physical and functional associations. Then to structure the signaling-metabolic interaction network (SMIN), cross-connections between the fourteen signal transduction and six groups of metabolic pathway proteins selected from pathway databases (see Materials and methods) were constructed based on protein-protein interacting proteins (node) and cross-connecting links/paths. All possible connections between any given signaling pathway protein (S) and metabolic pathway protein (M) via protein-protein interconnectors (PPIs) were included (Fig 2A). To derive a simplified but informative network, the interactions were restricted to the second level of protein interactors, i.e. up to interactors of interactors. This led to four different types of cross-connected paths where signaling pathway proteins were either directly connected with metabolic pathway proteins or through one, two or three PPIs, respectively (Fig 2A). These paths connecting all above-mentioned signaling (S) and metabolic (M) pathway proteins were then converted into networks based on the protein-protein interaction status between the involved proteins (Fig 2B).

**Fig 3:**
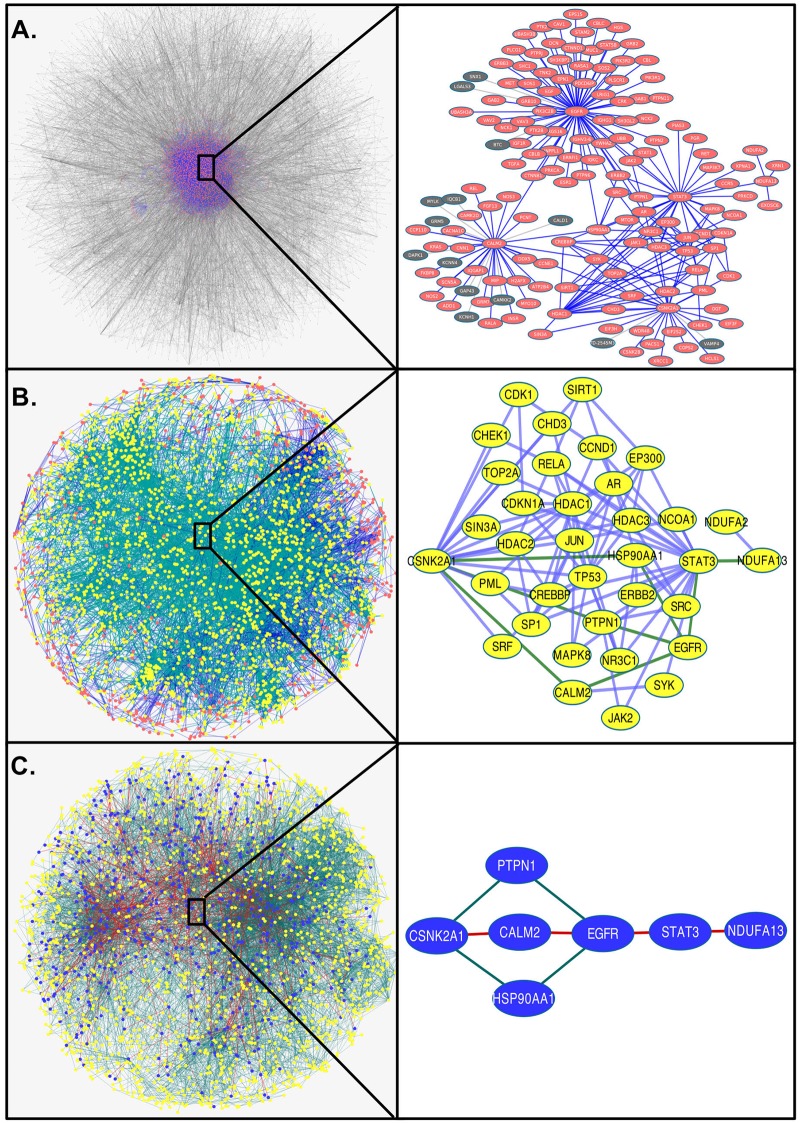
Development of a GBM-specific significant network from HPPIN. **(A)** A human protein-protein interactome network (HPPIN) was established based on experimentally validated interactome data of STRING database (left panel: the total network where nodes and edges are marked in grey). The SMIN was extracted from the HPPIN as signaling-metabolic interconnected path-dependent network between fourteen signaling and six groups of metabolic pathways as indicated in Fig 2 (highlighted as orange nodes and blue edges within the HPPIN on the left panel). The interconnections in the SMIN between one signaling (CSNK2A1) and one metabolic (NDUFA13) pathway protein through PPIs were highlighted on the right panel.**(B)** A GBM-specific network (left panel with yellow nodes and green edges) was developed from the SMIN (orange nodes and blue edges within the total background network) considering interconnecting paths with at least one differentially expressed protein identified from comparative proteome analysis of U87MG (EGFRwt) and U87MGvIII (EGFRvIII) GBM. The interconnecting paths between the same signaling (CSNK2A1) and metabolic (NDUFA13) pathway protein after exclusion of non-GBM specific interconnections are shown on the right panel. **(C)** The GBM-specific network based on significant nodes (z ≥1) and significant interconnected paths (path score ≥80% of the highest path score of each SM pair) identified from the weighted network were indicated with blue nodes and red edges within the GBM-specific network with yellow node and green edge. The interconnecting paths in the GBM-specific significant network between CSNK2A1 and NDUFA13 are shown as examples on the right panel.

The resulting signaling-metabolic interaction network (SMIN) is shown in the left panel of Fig 3A with nodes in orange and edges (connection between two nodes) in blue,within the total interactome containing HPPIN network with grey nodes and edges. As a result of afore-said restriction criteria, Fig 3A shows the reduction of the network size (number of nodes/proteins and their interactions/edges) of SMIN (11,059 interactions formed by 2,785 proteins) from HPPIN (16,828 interactions formed by 5,703 proteins). As an example of network links, the detailed connections and interactions between one signaling pathway protein, CSNK2A1, and one metabolic pathway protein, NDUFA13, through different PPIs in the SMIN are shown on the right panel of Fig 3A. These two selected examples are indicated by asterisks. The SMIN was found to contain 158 direct (S-M) linking paths, 4,036 with one interactor (S-P-M), 91,847 with two interactors (S-P-P-M) and 2,110,205 with three interactors (S-P-P-P-M). These paths were formed between 158, 2,967, 22,307, 69,032 S-M pathway protein pairs, respectively (Table 1). Comparisons with respective random networks proved that our selected HPPIN and SMIN are non-random scale-free networks (S1 Fig and S1 Table).

**Table 1:** Detailed statistics of paths, pairs, and proteins with expression involved in stepwise filtration of the signaling-metabolic interaction network (SMIN) to the significant GBM-specific network with the differential expression states in the EGFR-mutated cell line U87MGvIII compared to U87MG with wild-type EGFR.

Connection Types		Pairs and Paths							
		Supra Interaction Network (SMIN)		GBM specific Network		GBM specific paths and pairs in weighted network			
		Total S-M pairs	Total paths	GBM specific Pairs	GBM specific Paths	Model-1 Pairs selection		Model-2 Path selection	
								Z≥3	Z≥1
						Z≥3 pairs	Z≥1 pairs	≥80% Paths	≥80% paths
**S & M same**		51	-	2	-	-	-	-	-
**S-----M**		158	158	22	22	1	1	2	5
**S---P---M**		2,967	4,036	425	509	2	8	21	84
**S---P---P---M**		22,307	91,847	5,509	15,042	114	334	228	600
**S---P---P---P---M**		69,023	2,110,205	33,328	421,876	758	1,961	625	1,564
**Total**		69,352	2,206,246	33,418	437,449	801	2,055	876	2,253
**Total gene**		949	2,785	906	2,319	431	494	447	654
**Exp. status**	**UP**	17	48	17	48	11	15	17	22
	**DN**	24	85	24	85	8	12	23	32
	**NC**	53	162	47	142	11	19	24	40

### Expression of signaling and metabolic pathway proteins in GBM cell lines

To render the above condition-independent SMIN GBM-specific, quantitative comparative proteome analysis of the low- and high-grade GBM cell lines U87MG and U87MGvIII were performed and the data incorporated in the model to derive an enriched GBM-specific network. The use of cell lines helped to reduce the noise associated with the usual small size and heterogeneous cellular compositions of clinical samples. Proteomes was chosen as primary expression data to focus the models on the protein level, which is more closely related to the biological processes to be modeled than transcriptome data. Transcriptome date from clinical samples (see below) were subsequently added to reduce the missing data problem of proteomics and link the model to the clinical level. To decrease the complexity of the proteins in the cell extracts and minimize signal suppression by overabundant peptides, the proteins were first separated by SDS-PAGE. Then the gels were cut into one mm slices followed by separate in-gel digestion of the proteins with trypsin. The resulting fragments were extracted from the gel slices as individual samples, separated by reverse-phase nano-HPLC and analyzed on-line by ESI-Q-TOF mass spectrometry (S2 Fig). Quantification was done label-free by calculating the exponentially modified protein abundance index (emPAI) to avoid the drawback of mere signal intensity-based measurements. Only proteins with at least two tryptic fragments were identified by MS/MS with high confidence were considered, which further reduced the noise of protein identification although resulting in lower numbers of hits. The analyses were done with three independent replicates per cell line and the resulting data processed in two different ways. First, each of the three datasets for U87MG was compared to each of the three datasets for U87MGvIII resulting in nine pair-wise comparisons. Second, the average of three datasets of one U87MG was compared with the average of the three datasets for U87MGvIII.

**Fig 4:**
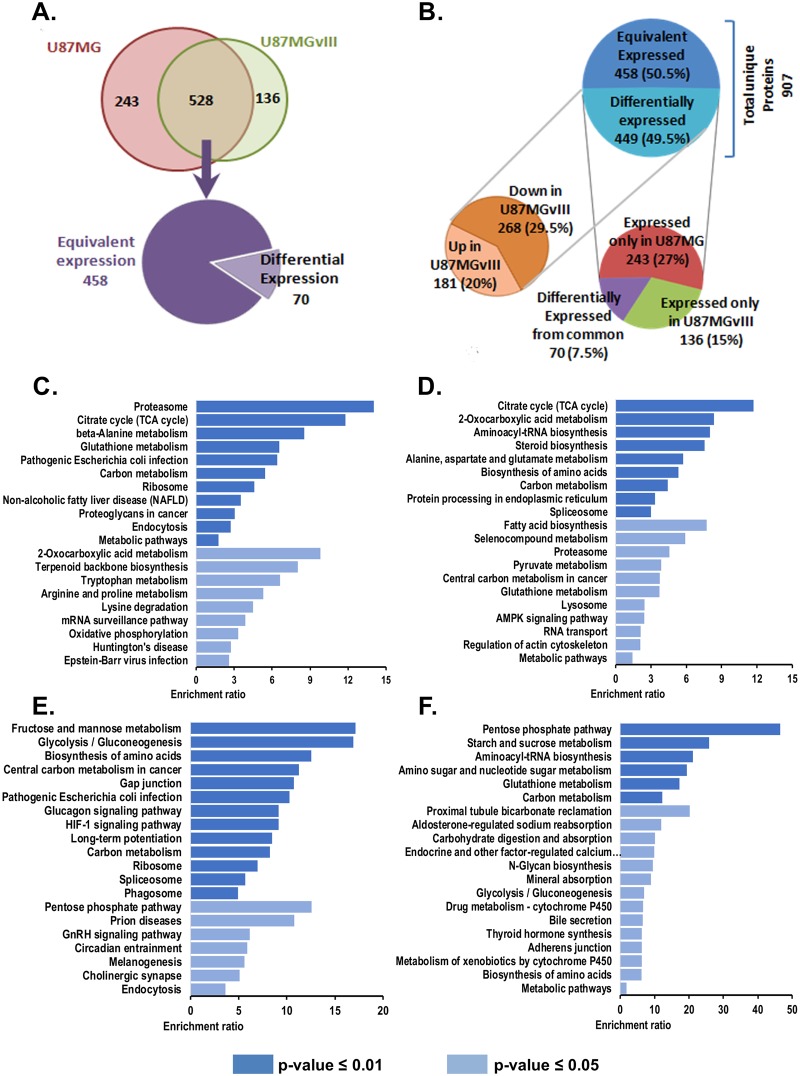
Comparative proteome analysis between the GBM cell lines U87MG (EGFRwt) and U87MGvIII (EGFRvIII). Protein identification and quantification were done by nano-HPLC Q-TOF MS/MS and peptide searches using the SwissProt databank for human proteins. Quantitation of the protein expressions was label-free by calculating the emPAI values for each protein. **(A)** Venn diagram for the statistics of proteins identified in U87MG and U87MGvIII. In three independent biological replicates of the proteome analysis for each cell line 907 proteins were identified, 528 common, 243 only for U87MG and 136 only for U87MGvIII cells. Of the shared proteins, 458 were equivalently and 70 differentially expressed. **(B)** Distribution of equivalently and differentially expressed proteins. Of the 449 differentially expressed proteins 243 were only present in U87MG and 136 in only U87MGvIII and 70 were present in both but in differential expression (down right). In total 268 were up-regulated and 181 down-regulated in U87MGvIII compared to U87MG. **(C-D)** Pathway over-representation analysis. Panels C and D plot the enriched pathways for proteins exclusively overexpressed in U87MGvIII (EGFRvIII) and U87MG (EGFRwt), respectively. One thirty four out of 136 and 235 out of 243 proteins overexpressed in U87MGvIII (EGFRvIII) and U87MG (EGFRwt) were mapped onto pathways. **(E-F)** Panels E and F plot enriched pathways of commonly up-regulated and down-regulated proteins between U87MGvIII and U87MG. Fourty five out of 45 and 24 out of 25 proteins commonly overexpressed in U87MGvIII (EGFRvIII) and U87MG (EGFRwt) were mapped onto pathways.

A total of 907 unique proteins were identified, 771 from U87MG and 664 from U87MGvIII. Five hundred twenty-eight proteins were expressed in both cell lines, 243 only in U87MG (EGFRwt) and 136 only in U87MGvIII (EGFRvIII). These distinctively expressed proteins were considered as overexpressed in the respective cell lines in comparison to the other. Compared quantitatively, 458 of the 528 common proteins were expressed at similar levels, 70 proteins were either up (45) or down (25) regulated in U87MGvIII compared to U87MG (Fig 4A). Together, nearly half of the identified proteins (449) were differentially expressed, 268 down-regulated and 181 up-regulated in U87MGvIII versus U87MG (Fig 4B).

Over-representation analysis (ORA) based enrichment for cellular pathways, biological processes and molecular functions were performed using cell line specific and commonly overexpressed proteins. Fig 4C and 4D show the top 20 most enriched pathways for proteins exclusively overexpressed in U87MGvIII (EGFRvIII) and U87MG (EGFRwt), respectively. Similarly, Fig 4E and 4F provide the top 20 enriched pathways for commonly over and under expressed proteins, respectively.

The most significantly enriched pathways were found to be proteasome (p-value = 0.97763E-07) in U87MGvIII (EGFRvIII) and TCA cycle (p-value = 1.48369E-06) in U87MG (EGFRwt). However, Fructose and mannose metabolism (p-value = 6.7086E-04) and pentose phosphate pathways (p-value = 3.1503E-05) were found to be most significantly enriched for proteins commonly overexpressed and underexpressed, respectively.

Gene ontology (GO) based biological process and molecular function over-representation analysis was also performed using cell line specific overexpressed proteins. Most significantly enriched biological processes and molecular function were found to be Tricarboxylic acid metabolic process (p-value = 2.7304E-04) and Threonine-type peptidase activity (p-value = 0.001053394), respectively in U87MGvIII (EGFRvIII). In U87MG (EGFRwt), TCA metabolic process (p-value = 7.76842E-08) and pre-mRNA binding (p-value = 0.007857451) were found to be the most significantly enriched biological process and molecular functions, respectively (S3 Fig).

### EGFR-mediated GBM-specific network

To uncover the signature of the reprogramming of global cellular processes by the EGF-independent constitutively active EGFRvIII in GBM, we mapped the data from the comparative proteomics of U87MGvIII (EGFRvIII) versus U87MG (EGFRwt) onto the above-described SMIN. All S-M interconnecting paths with at least one differentially expressed protein were extracted from the SMIN to generate a GBM-specific network (Fig 3B, left panel: highlighted with yellow nodes and green edges within the orange nodes and blue edges of the SMIN) with the assumption that those paths will have higher probability to be differentially active in mutant GBM condition. As an illustration of proteome data mediated extraction of a GBM-specific network, the interconnections between the aforementioned signaling pathway protein, CSNK2A1 and the metabolic pathway protein, NDUFA13 are highlighted after extraction from SMIN (Fig 3B right panel in comparison to Fig 3A right panel). Table 1 provides the details of the paths/pairs present at different stages of network development.

### GBM-specific network weighted for biological significance

To make this network further enriched with potentially disease-relevant paths/pairs, weights specifying disease-related biological properties and expression states of the proteins were assigned to each node (protein/gene) and edge (interaction) of the GBM-specific network. The following three categories of proteins (nodes) were given additional weights. First, proteins cross-talking between different signaling pathways (signaling cross-talk, SC), second, rate-limiting enzymes (RLE) for their roles in regulating metabolic rates and pathways, third, EGFR mutation-specific differentially expressed proteins (dEXP) for their GBM-specific impact. The GBM-specific network included 446 dEXP, 349 SC, and 267 RLE proteins. Of these, 11 SC and 17 RLE proteins were up or down regulated suggesting their involvement in signaling-metabolic cross-connection in EGFR-mutated condition (S4A Fig). For systems-level interpretation and understanding the network property, local signaling entropy (S_i_) was introduced. Previous studies [71–73] showed that S_i_ can be used as a measure of uncertainty in signaling information flow over a network and to identify important signaling pathways and genes/proteins in cancer. Effect-on-node (*eff*_s_) of every protein (node) in the network provided significance of a protein based on SC, RLE and dEXP in its local network. To identify probable paths of information flow from a signaling to metabolic pathways, network entropy (S_i_) and effect-on-node (*eff*_s_) properties were incorporated as node weights into the logic of the Hidden Markov Model (HMM). The edge weight of every two interacting nodes (gene/protein) were defined based on the principle of mass action (assuming that the probability of interaction of two genes in a given sample is proportional to the product of their expression values in the study samples) as probability of interaction (p_ij_, where i and j are the two nodes) in GBM condition. To assign the expression value of each node present in the GBM specific network, the average expression value of each gene was calculated from the normalized transcriptome data from 239 GBM patients. Incorporating these transcriptome data as edge weights linked the network with biological information from GBM patients. It helped to assign an extra weight other than previously mentioned node weight for all connections made by two nodes based on their expression in clinical GBM patients. Furthermore, it helped to add another level of constraint on over-prediction of information flow for nodes, which got an extra weight based on SC, RLE, and dEXP but are not expressed at higher levels in GBM patients. This helped to incorporate the contribution of those nodes to the disease, which were identified by neither of the three before-mentioned node weights nor by proteomics. Moreover, the much broader coverage of gene expression by genome-wide transcriptomics compared to proteomics helped to overcome some of the missing-data-problem of proteomic datasets.

**Fig 5:**
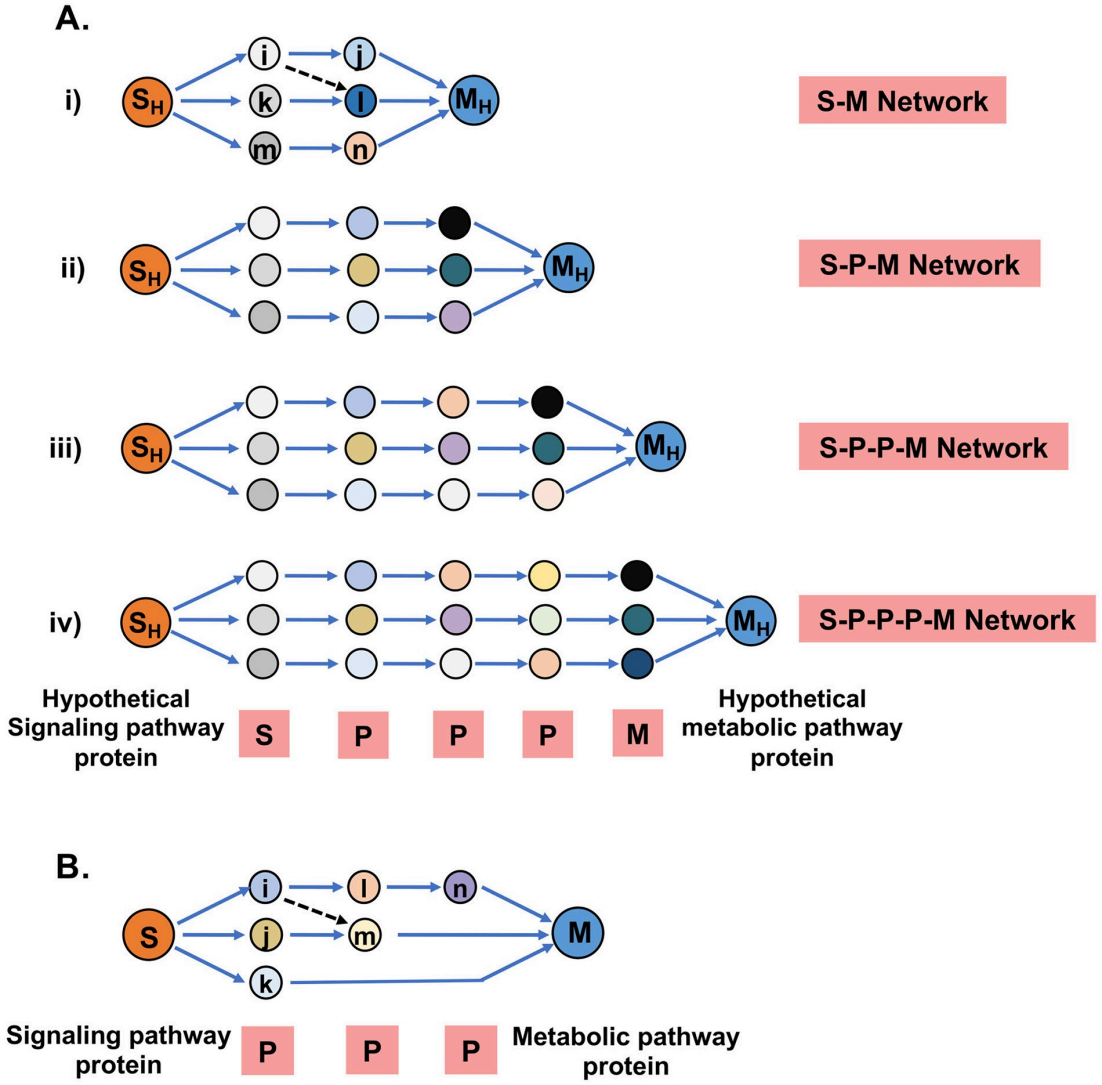
Models for the identification of significant signaling-metabolic (SM) pathway protein pairs and interconnecting paths. Models were implemented to identify significant SM pairs and paths from the weighted network. Weights of the nodes were defined based on whether the proteins were differentially expressed (dEXP), signal crosstalk (SC) proteins, rate-limiting enzymes (RLE) or none of these. The edge weights were defined as interaction probabilities derived from the gene expression values of two interacting proteins in GBM patients. **(A)** Model 1 was used to identify significant SM pairs from the GBM-specific weighted network. Four types of interconnection with none, one, two and three PPIs between S-M pairs were considered as separate models (i to iv). Two hypothetical proteins were considered on each side (S_H_ and M_H_) to implement positional probabilities based on the node weights of all proteins including S and M. Using these models and the Hidden Markov Model (HMM) logic, the path scores were calculated from the node and edge weights. The path scores were converted to Z-scores, and scores above the cut-off value of Z≥1 or Z≥3 were considered as significant and highly significant, respectively, and the respective interconnected SM pairs as significant pairs. **(B)** Model 2 was used to identify important linking-paths among the total possible paths formed between significant SM pairs from Model 1. Path scores were calculated from the positional probabilities as above. Higher path scores indicate higher significance.

An HMM-based simple mathematical formalism was used to understand context-specific information propagation from signaling to metabolic pathways in the human biological network. Node weights and edge weights were used to define the two major parameters of the Markov model, emission (E_f_) and transition (T_j_) probabilities, respectively. Two model systems were implemented to apply HMM logic, Model 1 for SM pair identification (Fig 5A) and Model 2 for S-M linking path identification (Fig 5B). Model 1 emphasized source (Signaling Pathway Protein, S) and destination (Metabolic Pathway Protein, M) pairs i.e. SM pairs having higher chances of information flow for each type of connections (Figs 2A and 5A). Model 2 was applied to find S-M linking paths between those selected pairs from Model 1. Selection of SM pairs (Model 1) and S-M linking paths (Model 2) was based on *Path*_score_ (see Methods for more details) a mathematical function of emission probability (E_f_) and transition probability (T_j_). For Model 1 positional emission probability was calculated considering the similar number of proteins because Model 1 was applied after grouping interconnecting links having the similar number of proteins forming the connection (Fig 5Ai–5Aiv). The calculated path scores of linking paths from the individual models were converted to statistical Z scores to identify the paths deviating from the mean. Based on the Z score under the individual models, the signaling-metabolic linking paths were classified as highly significant with Z score ≥3 (more stringent) or less significant with Z score ≥1 (less stringent) in EGFR-mutated GBM. The signaling and metabolic pathway proteins from the two ends of linking paths containing significant Z sores of each of the models were defined as the significant SM pairs. Multiple identifications of the same S-M pair from different models i.e. the formation of different types of significant linking paths involving different PPIs or different numbers of PPIs were nullified by considering them as a single. With that we identified 1, 2, 114 and 758 SM pairs meeting the more stringent cut-off (Z score ≥ 3) and 1, 8, 334 and 1,961 pairs with the less stringent cut-off (Z score ≥ 1) for the S-M, S-P-M, S-P-P-M and S-P-P-P-M linking types, respectively (Table 1, Model-1). In total 801 Z ≥ 3 and 2,055 Z ≥ 1 signaling-metabolic cross-connected SM pairs were identified between the 14 signaling and 6 groups of metabolic pathways as potentially important in EGFR-mutated GBM. These SM pairs were categorized according to the proteomic expression states of the source (S) and destination (M) proteins as UP-DOWN, UP-UP, DOWN-UP, and DOWN-DOWN. Including unidentified proteins in proteomics analysis (NA) of the cell lines, the couplet categories UP-NA, DOWN-NA, NA-UP and NA-UP, and unchanged expression states (NC) in the cell lines with SM categories UP-NC, DOWN-NC, NC-UP and NC-DOWN, and unidentified and unchanged SM pairs NA-NA and NC-NC were added (S4B Fig).

A number of cross-connections between signaling and metabolic pathways were identified with significant cutoff levels where either one or both pathway proteins (S and/or M) were not identified (NA) and/or unchanged (NC) by mass spectrometry indicating that the integrated network model can identify connections also where intermediate interactors are more important than SC, RLE or dEXP. The identification of pathway cross-connections is thus not dependent on proteomic identification of all constituent members but can be based on signaling crosstalk proteins and their expression status in GBM patients. It is important to include unchanged (NC) proteins in the model building as they might represent nodes in the paths that include other proteins that are differentially expressed. They might also play a role in the crosstalk between signaling paths and pathways or they might become important when known primary paths are blocked, e.g. by therapeutic intervention. The model could thus help to identify potential therapeutic targets for alternative therapies in cases of treatment failures or to design combination therapies that target primary together with potential escape pathways.

Mapping the pathway information of proteins in SM pairs showed which signaling pathways made a higher number of connections with which type of metabolic pathways in EGFR-mutated GBM. Six hundred one significant pairs with Z ≥ 1 were cross-connecting the MAP kinase pathway with all six groups of metabolic pathways (S2 Table). Similarly, the Ras, EGFR, AKT and p53 pathways were significantly connected to metabolic pathways through 570, 543, 549 and 179 SM pairs, respectively (S2 Table). As crosstalk between availabe signaling pathways is common whereas it is less common in between metabolic pathways (S5A Fig), some identified SM pairs and the respective proteins/genes may be shared. Analyzing the shared components in the five most connected signaling pathways revealed that MAPK pathway had the highest number of unique significant SM pairs (256) and genes/proteins (63) involved, followed by 194, 129, 116 and 95 unique significant SM pairs (S5B Fig) and 20, 30, 36, 19 genes/proteins (S5C Fig left) for the EGFR, AKT, p53 and Ras pathways respectively. Twelve cross-connected pathway protein pairs were common to all five pathways and 141 pairs shared by the Ras, EGFR, AKT and MAPK pathways indicating high connectivity between them, and their cross-connection with metabolic pathways suggesting important roles of the respective proteins in EGFR-mutated GBM (S5B Fig for pairs, C for genes left). In turn, the amino acid, carbohydrate, and nucleotide metabolic pathway groups were connected to all fourteen signaling pathways through 327, 289 and 326 cross-connected SM pairs (S2 Table, S5B Fig right)and 296, 260 and 268 genes in significant S-M paths, respectively (S5C Fig right). This indicates that altered cellular signaling related to the EGFR mutation and its constitutive activity affects most strongly these three metabolic pathway groups. As metabolic pathway enzymes interact via their substrates and products, there are few possibilities for interconnection between metabolic pathways except for the end steps, which is confirmed by the analysis of shared proteins. Twenty-three shared pairs were identified between the amino acid and the carbohydrate metabolic pathway, which relates to the low number of amino acids metabolites feeding into the tri-carboxylic acid cycle (S5B Fig right).

Multiple linking paths of different types (S-M, S-P-M, S-P-P-M, and S-P-P-P-M) or of the same type but through different PPIs were possible between SM pairs. Not all of these linking paths could be equally significant in EGFR-mutated GBM. To find the significant linking paths between the above-identified significant SM pairs, all possible paths between a single SM pair were considered under a single model (Model 2, Fig 5B). Since in Model 2 proteins forming interconnections between SM pairs vary, positional emission probabilities were calculated for these proteins. As an example, in Fig 5B the second position contained two proteins and third position one protein. Paths with path scores ≥80% of the highest path score for each SM pair were selected as significant from Model 2. Accordingly, all the significant paths were identified from high (more stringent Z ≥ 3) and less (less stringent Z ≥ 1) significantly specified SM pairs to identify the total of significant linking paths in the network. These analyses showed that 2, 21, 228, 625 and 876 significant paths were present with more stringent cut-off (Z ≥ 3) and 5, 84, 600, 1,564 and 2,253 paths with the less stringent cut-off (Z ≥ 1) for the four pathway types (Table 1). By these pathway-based analyses under less stringent condition (Z ≥ 1), 652 significant linking-paths were identified between 570 cross-connected SM pairs of the Ras pathway with all six groups of metabolic pathways (S2 Table). In addition, 668, 629 and 569 significant linking-paths were identified between 601, 549 and 543 cross-connected S-M pairs between the MAPK, AKT and EGFR pathways, respectively, and all 6 groups of metabolic pathways. Together, in EGFR-mutated GBM, these four signaling pathways were involved in the highest number of SM cross-connections with metabolic pathways: 368, 344 and 298 cross-connecting paths were found between the fourteen signaling pathways and 327, 326 and 289 cross-connected SM pairs involving the amino acid, nucleotide and carbohydrate metabolism, respectively (S2 Table).

### GBM-specific network based on significant SM pairs and paths

Based on the identified significant (less stringent Z ≥ 1) SM pairs and the significant linking paths (path score ≥80% of the highest path score of each SM pair), we converted (as in Fig 2B) the significant paths into a network (Fig 3C left panel: highlighted with blue nodes and red edges within yellow nodes and green edges of the GBM network). This filtered network is more specific for EGFR-mutated GBM conditions. The filtration further eliminated non-significant or unimportant SM pairs and PPIs, which is shown, as an example, for the interconnections between signaling pathway protein CSNK2A1 and metabolic pathway protein NDUFA13 (Fig 3C right panel compared to Fig 3B right panel).

**Fig 6:**
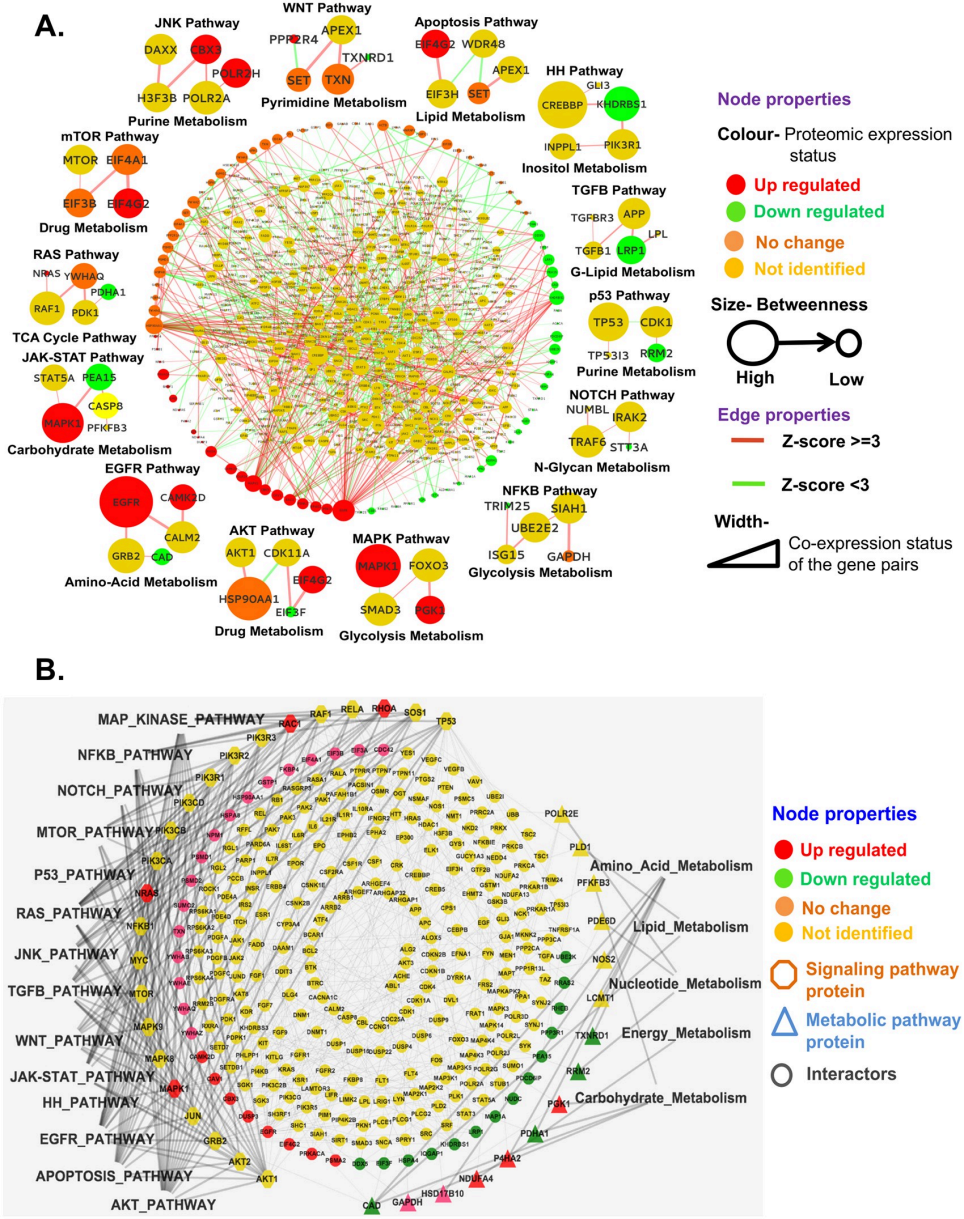
GBM-specific significant network. **(A)** The SM pairs and interconnecting paths between them that have a significant impact on EGFR-mediated GBM were filtered from the GBM-specific network using the mathematical model of Fig 5 and combined to generate the final signaling-metabolic interconnected network. The network parameters (color code of node and edge, node size, edge width mentioned in the figure) signified the cell-biological consequences of GBM driven by constitutive EGFR signaling. One representative signaling–metabolic connecting path for each of the fourteen signaling pathways is indicated at the side of the network.**(B)** Sub-network based on the 15 top-ranked signaling cross-talk proteins and their interconnection with all metabolic pathways.

The GBM-specific network based on significant SM pairs and linking paths was restructured to implement the biological consequences as network properties i.e. color-coded proteomic expression states (up, down, no change and not identified), size of node symbols proportional to the numbers of connections passing through it, colors of the edges as connection formed between more (Z ≥ 3) or less (Z ≥ 1) stringently defined SM pairs, width of the edge as the probability of interaction or product of the average expression values of two interacting genes in GBM patients from the transcriptome data (Fig 6A). The resulting network showed the important signaling pathways and their interconnections with metabolic pathways in EGFR-mutated GBM with the significance of every protein (size of the node) and their interactions with interacting partners (width of the edge). Around the network, representative paths between 14 signaling to metabolic pathways are shown as examples (Fig 6A, S6 Fig as more details of RAS pathway). To explore the importance of signal-crosstalk proteins in signaling to metabolic pathway interconnections in EGFR-mutated GBM, the sub-network dependent on the top fifteen crosstalk protein-based interconnecting paths were extracted from the GBM-specific significant network (Fig 6B). These sub-networks showed which signaling pathways are mostly cross-talking and how they are connected with metabolic pathways. This information was used to extract candidate genes/proteins/paths of EGFR-mutated GBM for further analysis.

### *In silico* perturbation test of the network

*In silico* perturbation analysis was performed for identification of paths that significantly change upon removal (e.g. by mutation or down-regulation) of a node (protein). To test the importance of the nodes/proteins in the final weighted network, each of the 654 nodes present in the Z ≥ 1 network (Table 1) was removed individually from the human interactome (HPPIN) and the node and edge weights were recalculated for the resulting networks and paths by recalculating Model 1 and Model 2 (Fig 2).

**Fig 7:**
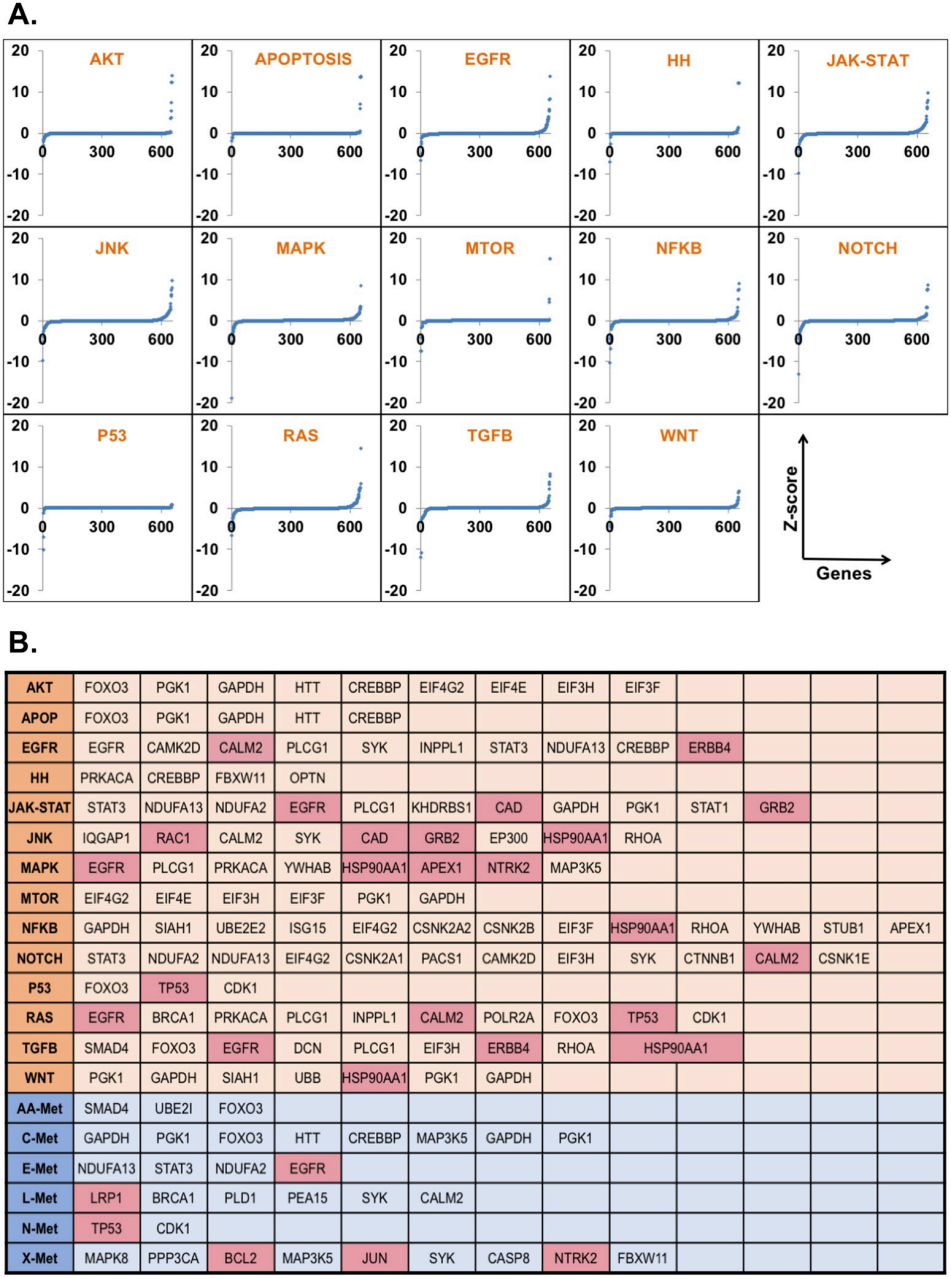
Importance of the individual nodes in signaling-metabolic pathway interconnection. **(A)** Each of the 654 nodes of the final network was tested in perturbation studies by removing it and its associated connections from the network and recalculating all network parameters. The scores for significant SM pairs (Z≥1) and interconnected paths (path score ≥80% from the highest path score for each SM pair) were redefined for each signaling to all metabolic interconnections. The differences of the sums of all significant path scores before and after perturbation of the individual 654 nodes for each signaling to all metabolic pathway interconnections were converted to Z scores (blue dots) and plotted against the genes/nodes. **(B)** Summary of the nodes with a significant impact on the entire network. Nodes with significantly altered Z score after perturbation (-2≥Z ≥2) were considered as effective nodes with significant impact and are listed in S3 Table for all interconnections of the 14 signaling pathways to the 6 metabolic pathway groups and *vice versa*, the metabolic pathway groups to the signaling pathways. Drugable node/proteins according to the KEGG database are highlighted.

Accordingly, new significant SM pairs (Z ≥ 3 or 1) were identified on the basis of Model 1 and significant paths (path score ≥80% of the highest path score) between them on the basis of Model 2. We mapped the pathway details of the SM pairs and calculated the average path scores before and after perturbation for the 14 signaling pathways to all 6 groups of metabolic pathways and *vice-versa*. The difference of values (before *vs*. after perturbation) for the 654 proteins for all pathways were converted to Z-scores and plotted for each perturbed node for each signaling pathway (Fig 7A). The nodes for which the Z-scores deviated from the mean as -2 ≥ Z ≥ 2 were selected as effective for the respective signaling pathway to all metabolic pathway interconnections in EGFR-mutated GBM (Fig 7B). S3 Table lists the numbers of significant and effective proteins identified for the individual signaling pathways connected to all metabolic pathways and from all signaling pathways to the individual metabolic pathways. As a measure of its effect on signaling-metabolic interconnection, each perturbed node was ranked according to the difference between baseline and perturbed condition. This means that highly ranked proteins have an important role in the connections of the respective signaling pathway to all metabolic pathways or *vice-versa*.

**Fig 8:**
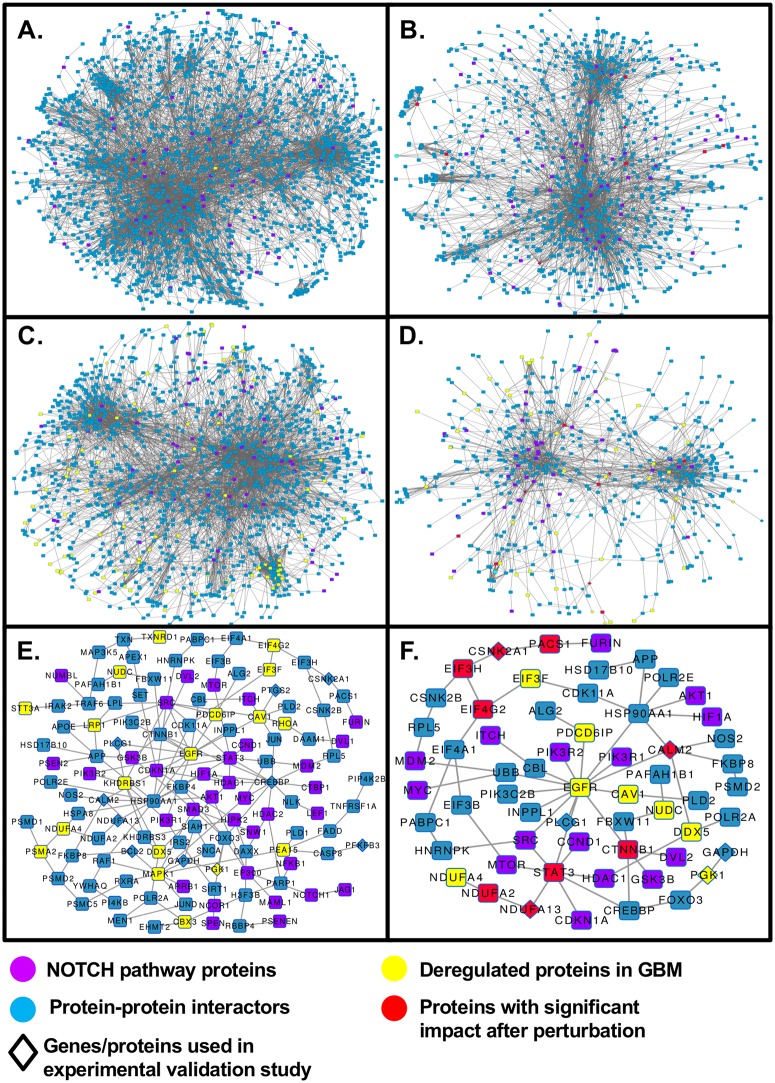
Enrichment of significant S-M interconnections by successive filtering during the development of the signaling-metabolic pathway network. The successive reduction of non-significant nodes and paths was followed for the inter-connection of the NOTCH pathway to all metabolic pathways. **(A)** Starting signaling-metabolic interaction network (SMIN) with the NOTCH pathway proteins in purple; **(C)** to GBM-specific network, and **(E)** the final GBM-specific significant (Z≥1) network. **(B)** Connections formed through twelve TOP Perturbation Impact (PI) nodes (red color) for NOTCH to all metabolic in SMIN; **(D)** connections formed through 11 nodes (in red color; out of 12 genes/proteins found with significant perturbation impact [PI]) in GBM-specific network and **(F)** connections formed through 9 nodes (in red color; out of 12) in the GBM-specific significant (Z≥1) network.

The NOTCH pathway is shown as an example for the level of reduction in the network size when the GBM network is transformed to the significant GBM network to identify significant interconnecting paths and proteins (Fig 8). Fig 8A shows the interconnections of NOTCH pathway proteins with all metabolic pathway proteins present in the signaling-metabolic interaction network (SMIN) and Fig 8B shows only those NOTCH pathway proteins with interconnections to metabolic pathway proteins passing through effective nodes, i.e. nodes identified by the perturbation experiments (Fig 7B). Fig 8C presents the interconnections of the NOTCH pathway to all metabolic pathways in the GBM-specific network filtered on the basis of the weightage parameters for the nodes and edges, and Fig 8D shows the interconnections between the significant proteins only. Fig 8E shows the interconnections of the NOTCH pathway to all metabolic pathways in the GBM-specific significant network and Fig 8F the same only for significant nodes. The comparison of Fig 8B, 8D and 8F on basis of the effective node identified by the perturbation study (red colored) indicate the levels of filtration from the starting SMIN to the final GBM-specific significant network. We found 457,111 paths involving 1941 genes/proteins and 8047 interactions out of a total of 2,206,246 paths in the SMIN connecting the NOTCH signaling pathway to all metabolic pathways, of which 10% were found to have a significant (Z-score cut-off -2 ≥ Z ≥ 2) perturbation impact (PI) (Fig 8B). Comparable reductions of approximate 75% (Fig 8C) and 50% (Fig 8D) for both nodes and interaction were found when going to the GBM-specific condition. After *Path*_score_ based filtration (Z-score ≥ 1), 146 paths involving 125 nodes and 166 interactions were identified in the NOTCH pathway (Fig 8E) of which 51 paths involving 59 nodes and 65 interactions formed by nodes with significant PIs.

### Experimental validation of signaling-metabolic network

**Fig 9:**
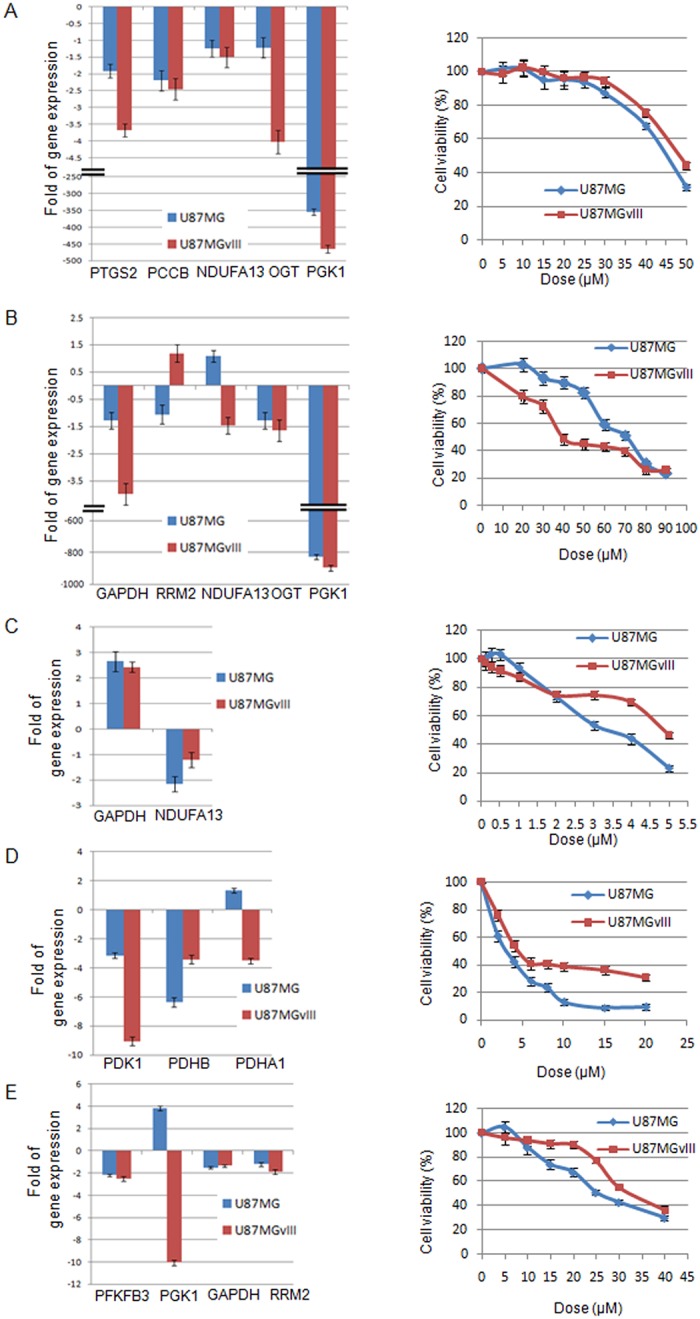
Test of the GBM-specific significant network model in cell cultures. The EGFRwt and EGFRvIII-mutated GBM cell lines U87MG and U87MGvIII were incubated with inhibitors for calmodulin (CALM2) with CGS 9343B **(A)**, casein kinase II subunit alpha (CSNK2A1) with Emodin **(B)**, 1-phosphatidylinositol 4,5-bisphosphate phosphodiesterase gamma-1 (PLCG1) with U73122 **(C)**, tyrosine-protein kinase ABL1 with Dasatinib**(D)**, and B-cell lymphoma 2 (BCL2) with ABT-199 **(E)**. The bar diagrams on the left show the effects of the inhibition of the expression of metabolic pathway proteins predicted to be interconnected to the targeted signaling pathway proteins. The line graphs on the right show the dose-dependent effects of the signaling protein inhibitors on the viability of the cells.

The result of perturbation study proved the importance of the respective nodes in the final network for information flow from signaling to metabolic pathways. To validate these findings of interconnections between signaling pathway alterations and metabolic rearrangement, some of SM connections were selected from the final network for experimental validation (Fig 9). The selection was based on the effect of the removal of the respective node in the *in silico* perturbation experiments, the predicted effects on the metabolic pathways and the availability of specific small-molecule inhibitors, excluding transcription factors as their impact is obvious. The selected targets were calmodulin (CALM2), casein kinase II subunit alpha (CSNK2A1), 1-phosphatidylinositol 4,5-bisphosphate phosphodiesterase gamma-1 (PLCG1), the tyrosine-protein kinase ABL1 and B-cell lymphoma 2 (BCL2). The signaling-metabolic interconnecting paths including the selected proteins were extracted from the final GBM-specific significant network using the most stringent cutoff and the connected metabolic proteins linked to the above-mentioned signaling pathway proteins were identified (S1 File). As the translation of oncogenic signaling to metabolic adjustment is via the expression of metabolic enzymes, the changes of expression of the metabolic pathway proteins upon inhibition of the selected signaling proteins with small molecule inhibitors and cell viability were analyzed. The inhibitors were CGS-9343B for calmodulin (Fig 9A), Emodin for CSNK2A1 (Fig 9B), U73122 for PLCG1 (Fig 9C), Dasatinib for ABL1 (Fig 9D), and ABT199 for BCL2 (Fig 9E). U87MG (EGFRwt) and U87MGvIII (EGFRvIII) cells were incubated with the inhibitors and their effects on the expression of the predicted interconnected metabolic pathway proteins analyzed by quantitative RT-PCR, and the viability of the cells tested in MTT assays. The blockade of the signaling pathway proteins had significant impacts on the expression of the metabolic pathway proteins (Fig 9). In the case of the calmodulin inhibitor, the expression levels were reduced in both cell lines but for prostaglandin-endoperoxide synthase 2 (PTGS2) and *O*-linked β-*N*-acetylglucosamine transferase (OGT) much more pronounced in the EGFR-mutant cell line than in the wild type. The strongest effect was seen for phosphoglycerate kinase 1 (PGK1) with around 400-fold reduction of expression in both cell lines. The effects of the CSNK2A1 inhibitor are more differentiated. While the OGT and PGK1 expression is inhibited uniformly in both cell lines, 800-fold in case of PGK1, the inhibition of glyceraldehyde 3-phosphate dehydrogenase (GAPDH) is much stronger in U87MGvIII than in the wild type, the expression of ribonucleoside-diphosphatereductase 2 (RRM2) is decreased in the wild-type and enhanced in the mutant, and that of NADH dehydrogenase [ubiquinone] 1 alpha sub-complex subunit 13 (NDUFA13) is affected inversely. Inhibition of phospholipase C gamma 1 (PLCG1) results in enhanced expression of GAPDH and reduced expression of NDUFA13. The ABL1 inhibitor reduced the expression of pyruvate dehydrogenase kinase subunit 1 (PDK1) in both cell lines but much more in the mutant cells. In contrast, the expression of pyruvate dehydrogenase (lipoamide) beta (PDHB) is more reduced in the wild type, and that of pyruvate dehydrogenase subunit alpha 1 (PDHA1) is slightly enhanced in the wild-type and reduced in the mutant cells. Finally, the BCL-2 inhibitor reduces the expression of 6-phosphofructo-2-kinase/fructose-2,6-biphosphatase 3 (PFKFB3), GAPDH and RRM2 2-fold in both cell lines but enhances the expression of PGK1 in the wild-type 4-fold while reducing it in the mutant cells 10-fold.

All signaling pathway inhibitors have pronounced negative effects on the viability of both GBM tumor cell lines (Fig 9) but U87MGvIII is significantly more sensitive to the inhibition of casein kinase than the wild-type whereas the wild-type is more sensitive to the inhibition of PLCG1, ABL1 and BCL2. Half-maximal reduction of viability is seen for the PLCG1 and the ABL1 inhibitors in the single-digit, for the others in the double-digit micromolar range.

Further, the effect of the signaling molecule inhibitors on cell migration and invasion was tested. The results showed the similar negative effects of the inhibitors with a higher sensitivity of U87MGvIII, which reconfirms the potency of the signaling-metabolic interconnected network model (S7 Fig).

## Discussion

In all cellular condition, signaling, gene expression, and metabolic pathways must be coordinated to maintain cellular integrity and functions [74]. In this study, we propose an integrative systems biology model of information flow between signaling and metabolic pathways that use an interconnected scaffold based on the complete set of human interactome (HPPIN). The model successfully detects probable unique connections of genes/proteins involved in the interconnection of different signaling and metabolic pathways. The identified components of the network (i.e. genes/proteins as nodes and connections through edges) represent known physical and/or functional associations between proteins/genes. Depending on the oncogenic signaling, the interconnectors involved modulating the metabolic pathways change. We have successfully applied the model to identify the interconnections altered in the constitutive signaling of the mutated EGFR in glioblastoma multiforme (GBM) compared to EGF-dependent and wild-type EGFR (EGFRwt) signaling.

So far, the development of integrated models including all three or any two of signaling pathways, gene regulation and metabolic pathway in any cellular context is still very restricted. Difficulties of integration arise from the mode of information flow in these three processes significantly involving activation/inactivation, inhibition/induction and substrate/product respectively, as well as different extents and time frames or kinetics [74]. Attempts to integrate metabolism and gene regulatory networks were based on regulatory flux balance analysis (rFBA) [75,76], steady-state regulatory FBA (SR-FBA) [77], probabilistic regulation of metabolism (PROM) [78], integrative omics-metabolic analysis (IOMA) [79] and ordinary differential equation (ODE) [80]. There is a straightforward interconnection between signaling and gene regulation through transcription factor (TF) but the little informative interface and the dynamics of cellular localization of TF affect the integrative modeling. A few studies addressed the issues using the integrative logical, influence graph, Boolean and thermodynamics models [81–86]. Attempts to integrate signaling and metabolism is rarer because information in the two process areas flows in different ways via activation/inactivation and substrate/product interactions respectively. The few reports that address this issue are based on ODE, combined dynamic ODE and Boolean models [33, 80, 87]. Here we have taken a novel approach and integrated signaling and metabolic pathways by i) creating a weighted PPI network depending on the path of information flow from signaling pathway molecule to metabolic enzymes, and ii) applying the probabilistic framework of Markov processes to calculate information flow scores. We have used two major functions of the Markov model. First, to estimate the probability of a protein to be present at a particular point in the network to transmit information, transition functions (node weights) were implemented. Second, to estimate the connection strength between two proteins, emission functions (edge weights) were introduced. The two functions utilize different biological parameters to explore the integrated network and to calculate S-M *Path*_score_. In contrast, to other models for integration of signaling and metabolism pathways, the comparative proteomic expression of the signaling molecule, metabolic enzymes and interactors are effectively incorporated to construct a weighted PPI network (one criterion of node weights) and to filter out context-dependent (mutated vs. wild-type EGFR GBM) S-M interconnections. The second attempt toward integration of the two pathway types to make modeling information flow more accurate and render the model context-dependent based on patient-related disease-specific parameter, trancriptomic expression value from GBM patients were implemented for each node and, by using the principle of mass action, edge weight were defined. The implementation of these two layers of information distinguishes the model from others by integrating weighted network properties like network entropy (S_i_) and effect-on-node (*eff*_s_). We believe that these have the potential to capture context-dependent properties of PPI networks in terms of information flow [88]. Genes/proteins having a high impact on the path formation are important in their local network as well as the global context. Unlike considering conventional network centralities (hubs, bottlenecks etc.) of nodes, biological state and expression profiles are used.

Integrating proteome and transcriptome data in the development of the model helps to mitigate problems in translating high-throughput omics into systems biology models. While proteome data more closely related to the biomedical and pathological systems properties of interest, they are less complete and more prone to variation and experimental errors then transcriptome data. The use of proteome data from cell lines helps to some extent to overcome the experimental problems, however, cell lines are selected cell culture-adapted models and may be quite remote from *in vivo* situations. Transcriptome data from clinical specimens are getting increasingly available and may add a close-to-clinic dimension to the systems biology model, and help to overcome the missing-data problem of proteomics. Furthermore, the two omics data sets were used at different steps of the network establishment and for different biological relevance. The proteome data of the EGFRwt and EGFRvIII-mutated cell lines were used first to filter out the GBM-specific network from signaling-metabolic interaction network (SMIN). On that GBM-specific network, we have incorporated the human GBM patient transcriptomic data as edge weights to make it more relevant to human GBM condition.

The resulting model is probabilistic, based on the assumption that the likelihood of signal flow relates to the expression levels of the nodes/proteins in the different paths/pathways. It is not computing the actual signaling status of the paths and their nodes. Incorporation of phosphoproteome data in this integrated network could provide additional information about the activation/deactivation state of the nodes thus adding elements of the actual information flow. This could help to add some rationale weights for nodes that remained unchanged (NC) or were unidentified (NA) in the proteomic study as well as in GBM patient transcriptome data. Accordingly, it could add to solving the missing-data-problem of proteomics. Although phosphoproteome data could provide relevant information about signaling flow it could be less advantageous in the model of interconnection between the oncogenic signaling and metabolic pathways that are required to sustain the oncogenic processes. It could make the model more complex as expression and phosphorylation of proteins are not correlated directly; phosphorylation states are highly context- and time-dependent and requires to take into account the ratio of phosphorylated and non-phosphorylated signaling proteins at any given position within the cell and the spatial arrangements of active/inactive signaling pathway molecules.

To test the network properties, the dynamic behavior and the robustness of the model, we performed *in silico* perturbation experiments. The *in silico* perturbation experiments were done with the final GBM-specific model with all interactome, proteome and transcriptome data incorporated in this order to render a general interactome map GBM-specific, and calculate node/edge weights and path score. *In silico* perturbation was done by silencing the signaling molecules one by one, and then redeveloping the model and recalculating node/edge weights and path scores to determine the relevance of the respective signaling molecule and the affected signaling path, to identify possible escape paths bypassing the blockade, and to identify targets for experimental validation of the model. Based on the perturbation analysis we postulated that proteins with higher impact as loss-of-function or gain-of-function or *Path*_score_ difference between signaling-metabolic pathways and *vice-versa* have a higher ranking and chances to transmit information between the studied pair of pathways. The perturbation studies also captured the weighted topological changes in the dynamic network and the distinct sets of interactors that transmit information between proteins. Implementation of path score differences before and after perturbation adds additional accuracy to the model to identify the most important paths of information flow among all probable paths. Applying this model from 437,449 probable GBM-specific S-M paths, we identified 2,253 (Z≥1) (0.52%) and 876 (Z≥3) (0.20%) significant paths of information flow in mutated EGFR-dependent compared to wtEGFR EGF-driven GBM. To experimentally test and validate the interconnections predicted by the model, inhibition of signaling pathway proteins (S) present in the SMIN with small molecule inhibitor was performed, which resulted in alteration of metabolic pathway protein expression (M). We found that the performance of the model in predicting the dynamics of the large-scale signaling networks is comparable to state-of-art methods for extracting context-dependent information flow [89]. One node knockout at a time in the *in silico* perturbation experiments indicated, first, the importance of that protein in the signaling-metabolic interconnected paths in establishing low or high grade GBM condition, and second, alternative signaling routes that can become relevant as escape responses to therapy.

It is important to note that in biological networks including signal transduction, PPI is highly dynamic in nature and may undergo continuous changes [90]. These context-specific network dynamics need to be predicted by systems biology models. Our probabilistic integrated network model captures network dynamics using the topology of PPI networks based on dynamic data under different circumstances. The probabilistic network-based dynamic model uses experimental data and deals with the uncertainty of systems to predict drug targets and understand the effect of therapeutics. In our study, nodes forming paths significant to information flow in a particular biological context is the key to network inference. Nodes present in information flow path with high network weights and connected with higher edge weight can be considered for drug intervention and combinatorial therapy. Our model efficiently identified key nodes which are important in S-M information flow with confirmation of some known and prediction of the potential novel drug targets [89] that can be used alone or in combination to inhibit mutated EGFR-mediated GBM.

This model is now a useful tool and will be used to develop strategies and experiments to study causal relationships. Among the questions to be addressed will be to understand the molecular biology of high-grade GBM versus low-grade GBM, and mechanisms of therapy resistance. However, in its current form, this model is not engineered to measure signal flow in and across signaling pathway(s). It is also not applicable to other regulatory mechanisms such as transcriptional, post-translational and miRNA based modulations of biomolecular interactions. Nonetheless, the model should be robust enough to integrate and utilize large-scale pan-omics data including genomics, transcriptomics, proteomics, metabolomics and post-translational modification information to accurately model the cellular context under a given scenario. These issues are important for assessing systemic changes and we are addressing them in follow-up studies. The strategy for developing the model can also be applied to other cancers and to non-oncological conditions.

Interestingly, GAPDH and PGK1, commonly reported reference [91] and housekeeping genes [92], were identified (Fig 7B) as significant (~40% of 20 networks in the study) by our model and previously reported studies [47] have shown their association with cancer [93–95]. In this study, also significant expression change in two cell lines for GAPDH and PGK1 was found after inhibition of CSNK2A1 and BCL2, respectively.

To our knowledge, this has not been reported before. A study [96] with a mice model has shown increased expression level of *bcl-2* under the control of *pgk*-1, which suggests a possible relation to our findings. Not surprisingly, we have also found FOXO, a key player in cell fate decision [97] as candidate target. In addition, the HSP90 family gene (HSP90AA1) and CREBBP were identified as important agents in forming interconnections between multiple signaling/metabolic pathways. Inhibitors of HSP90 (17-AAG) and CREBBP (ICG-001) have recently shown effects in glioblastoma and other cancer models/cell lines [98–103]. They have entered clinical trials for several cancer types [104, Clinical trial numbers:NCT01606579, NCT01764477, NCT02413853]. This confirms that our model can identify potential therapeutic targets before performing actual drug tests.

The presented network model can be used to explore novel therapeutic strategies against cancer including combination therapies. This work also represents a step towards finding alternative routes/pathways in cancer or other diseases and thereby predicting the potential path of therapy evasion that can be included in the development of new therapies that aim to prevent therapy resistance in cancer and other diseases.

## Materials and methods

### Cell lines and cell culture

The human grade-IV glioblastoma cell line U87MG expressing wild-type EGFR (U87MG) was purchased from ATCC (USA). The U87MG-derived genetically engineered U87MGvIII cell line with exons 2–7 deleted from the EGFR gene (EGFRvIII) cell was a kind gift from Professors Webster K. Cavenee and Frank B. Furnari, Department of Medicine and Cancer Center, University of California at San Diego, La Jolla, USA. The cells were grown in Iscove’s Modified Dulbecco’s Medium (IMDM; Gibco, Thermo Fisher Scientific Inc., Schwerte, Germany) supplemented with 10% heat-inactivated fetal calf serum (FCS; Biochrome, Berlin, Germany) and 1% penicillin/streptomycin solution (Gibco, Thermo Fisher Scientific Inc., Schwerte, Germany) in a humidified atmosphere with 8% CO2 at 37°C.

### MTT assay

GBM cells (1 × 10^4^) were plated in 96 well plates and treated with inhibitors of Calmodulin (CGS-9343B, Sigma), Casein kinase II subunit alpha (Emodin, Sigma), 1-phosphatidylinositol 4,5-bisphosphate phosphodiesterase gamma-1 (U73122, Sigma), Tyrosine-protein kinase ABL1 (Dasatinib, Santacruz) and B-cell lymphoma 2 (ABT199, Santacruz) for 24hr and subsequently incubated with 3-(4, 5-dimethylthiazol-2-yl)-2, 5-diphenyl tetrazolium bromide (MTT,100 μg/ml DMSO) in fresh culture medium for 3 hr. Optical density (OD) was taken at 550 nm with an ELISA reader (Thermo) as described elsewhere [105].

### Scratch wound migration assay

U87MG and U87MGvIII were plated separately in 6-well plates with>90% confluence. Scratch-wounds were made with a micropipette tip, washed thrice to remove the floating cells and treated separately with ABL1, PLCG1, BCL2, CALM2 and CSNK IIA inhibitors at their IC50 dose (5μM, 3μM, 25μM, 45μM and 40μM, respectively) in medium and incubating them for the indicated time periods. Images were taken at 0 hrs and 8 hrs. The wound width was measured for untreated and treated groups from at least five different fields of three separate experiments, and percentage wound healing was calculated from the width at 8 hrs versus the initial width at 0 hrs, all using ImageJ software.

### Invasion assay

U87MG and U87MGvIII cells (5x10^4^) were seeded separately to the upper part of matrigel-coated invasion chamber in serum-free medium (200 μl) and the lower chamber was filled with medium (600 μl) containing respective inhibitors of ABL1 and PLCG1 at their IC50 doses. The cells on the lower surface of the insert were stained with the crystal violet after 24 hrs and the numbers of invaded cells were counted by inverted light microscopy from at least three different fields of three different experiments.

### Expression analysis by real-time PCR

U87MG and U87MGvIII cells were treated with the indicated inhibitors at half of the IC_50_ dose for 24hr. Total mRNA was isolated by RNeasy Mini Kit (QIAGEN) as per manufacturer’s instructions. RNA (1.0 μg) was reverse-transcribed to cDNA with the Reverse Transcriptase kit (Promega). For RT-PCR, the cDNA was amplified with specific primers for the mRNA of the indicated metabolic proteins in a Perkin-Elmer DNA thermal cycler. Real-time PCR analysis was performed by mixing cDNA with 2× SYBR green master mix using Roche Applied Science light cycler 480.0 instruments with the software version 1.5.0. Relative quantification of each target gene was normalized to two housekeeping genes (18s rRNA and β-actin) and expressed as a fold change compared with untreated control using the comparative cycle threshold (CT) method [6].

### Protein preparation

For proteome analysis, total proteins were extracted with SDS-PAGE sample buffer from U87MG and U87MGvIII cells grown to 80% confluency. EGFR expression status of both cell lines was confirmed by western blot analysis. Proteins (100 μg) of each cell line were separated by SDS-PAGE (10% acrylamide/0.8% bisacrylamide). After staining with Coomassie blue, the lanes for both cell lysate were sliced into one-mm thick gel pieces. The slices were transferred into 96 well round bottom polypropylene plate and destained with 100 μl, 20 mM ammonium carbonate/acetonitrile (60%/40% v/v), dehydrated by adding twice acetonitrile (50 μl) and dried under vacuum in a speed vac. The dried gel slides were rehydrated in 20 μl trypsin solutions (10 ng/μl) in 30 μl NH_4_HCO_3_ (20 mM) for digestion by incubation for 16–18 hr at 37°C. The supernatants were collected in 1.5 ml reaction tubes and the remaining tryptic fragments extracted from the gel slices first with 50 μl 50% acetonitrile with 0.1% TFA for 15 min, and then with 5% acetonitrile with 0.1% TFA. The extracts from each gel slice were combined and dried in a speed vac. The peptides were re-dissolved in 12 μl 2% acetonitrile with 0.05% TFA.

### LC-MS/MS

For MS/MS, the peptides of the tryptic digests (10 μl) were loaded via a pre-column at a flow rate 20 μl/min (2% acetonitrile, 0.05%TFA) onto an Acclaim PepMap C18 nano-HPLC column (75μm inner diameter×15 cm length; Thermo Fisher Scientific Inc., Schwerte, Germany) using an Ultimate 3000 nano-HPLC system (Dionex, Darmstadt, Germany). The peptides were eluted with a gradient of 5–60% solvent B (0.1% formic acid in 95% acetonitrile/5% water) in solvent A (0.1% formic acid in 1% acetonitrile/water) over 60 min, followed by 60–90% solvent B over 5 min, and 90% solvent B for 5 min at a flow rate of 220 nl/min. The nano HPLC system was directly coupled to a Micro-TOF-Q I mass spectrometer (BrukerDaltonics, Bremen, Germany). Mass spectra were acquired in the m/z range 50–2500 at an acquisition rate of 1.3 per sec. MS/MS spectra were acquired in the data-dependent mode with the fragmentation of the 5 most intensive peaks (absolute threshold 3000) with argon as collision gas, and fragment masses in the range of 400–1400 m/z. The mass spectrometry was run with dynamic exclusion of a time interval established and tested beforehand to avoid or minimize signal suppression by over-abundant peptides. A dynamic exclusion of 1 min was set to avoid repeated fragmentation of the most abundant peptides.

### MS/MS data analysis

The MS and MS/MS spectra were processed with Data Analysis 3.4 and Biotools 3.1 (BrukerDaltonics). MS/MS searches for peptide identification were done via Biotools on a local Mascot (version 2.2) server with a precursor mass tolerance of 50 ppm and a fragment mass tolerance of 0.2 Da. Trypsin was specified as an enzyme, and one allowed missed cleavage and oxidation of methionine as variable modification. Data searches were done in SwissProt databank for human proteins. Proteins were identified with at least two peptides with a mascot score of higher than 26. The false discovery rate with these search and filter parameters was below 5% as confirmed with a decoy database. Exponentially modified protein abundance index (emPAI) was employed for protein quantification using the equation

$$emPAI=10^{\frac{N_{observed}}{N_{observable}}}-1$$ (i)

where N_observed_ is the number of experimentally observed and N_observable_ the calculated number of observable peptides for each protein [106]. Comparative proteome analysis was done with proteins identified by at least two peptides based on the emPAI scores for differentially expressed proteins. The proteome analysis was done three times for each cell line as an independent biological replicates.

### Comparative proteomics of U87MG and U87MGvIII

Comparative proteome analysis was done for each proteome dataset (batch) of U87MG with each batch of U87MGvIII using the in-house PROTEOMESTAT-12 software. For this, the emPAI values of all proteins were normalized as

$${emPAI}_{norm}=\frac{{emPAI}_{protein}}{{\left[{\sum emPAI}_{protein}\right]}_{batch}}\times 100$$ (ii)

where *emPAI*_*protein*_ was the emPAI value of a protein in a batch.

Multiple identifications of a protein within a proteome dataset were excluded, only keeping the highest emPAI value for subsequent analysis. Intra-batch analysis was performed to determine the total unique proteins as well as the overlap between the three batches of each cell line. Total unique proteins identified from U87MG and U87MGvIII cells were compared to identify the common and the differentially expressed proteins. Proteins identified only for one of the cell lines were considered up-regulated in that cell line. Commonly expressed proteins were further analyzed in two different ways to identify differentially expressed proteins. In one approach, the emPAI_ave_ values for each protein were compared between the two cell lines (iii).

$${emPAI}_{ave}=\frac{\sum_{batch=1}^{3}{emPAI}_{protein}}{Number\;of\;batches\;with\;identified\;protein}$$ (iii)

where *emPAI*_*protein*_ was the emPAI value of a protein in a proteome dataset.

The percentages of up or down-regulation of a protein in U87MGvIII were calculated as *% Exp*_U87MGvIII_ using the following the equation

$${\%Exp}_{U87MGvIII}=\frac{\left({emPAI}_{ave\;U87MGvIII}-{emPAI}_{ave\;U87MG}\right)}{{emPAI}_{ave\;U87MG}}\times 100$$ (iv)

where emPAI_ave U87MGvIII_ and emPAI_aveU87MG_ were the average emPAI value of a protein among the three proteome datasets for U87MGvIII or U87MG. Proteins that were ≥ 50% up- or down-regulated with p values ≤ 0.2 were considered differentially expressed in the U87MGvIII cell.

In a second approach, emPAI_norm_ of a protein of each batch of one of the cell lines were compared separately with the emPAI_norm_ for the same protein in each of the three batches of the other cell line. Then these comparative data were normalized by the LOESS method. Up- or down-regulation of a protein in U87MGvIII were calculated using the Eq (iv). From this second approach ≥ 50% up- or down-regulated proteins with an SE ≤ 35% were taken as differentially expressed in U87MGvIII. Both the results were combined and duplicates were excluded to identify the differentially expressed proteins.

### Human signaling-metabolic interaction network

A signaling-metabolic cross-connected network was created using 14 signaling and 81 metabolic pathways and protein-protein interactors of the pathway proteins. Signaling pathway databases were created by integrating information from on-line resources including KEGG [107], Reactome [108] Signallink [109], NetPath [110] and Biocarta (http://www.biocarta.com) for extensive coverage of the steps involved in the pathways. The 14 signaling pathways were included based on their association with cancer in general according to KEGG pathway database.

Metabolic pathway datasets were collected from KEGG. Human protein interaction data were taken from the STRING interactome database using only protein-protein interactors [70] established by high-throughput analyses with the high confidence score (≥ 0.7) to generate a protein-protein interaction network (PPIN). PPI were restricted up to the second level interactors, i.e. interactors of interactors, which brings about S(PPI)_3_M, meaning S-PPI_1_-PPI_2_-PPI_3_-M paths. PPI_1_ is the first level interactor of signaling molecule and PPI_3_ is the first level interactor of metabolic enzyme and PPI_2_ the second level interactor of both. Thereby, there are three PPIs between the signaling and the metabolic pathway proteins. The resulting network consisted of 5,703 proteins with 16,828 experimentally confirmed interactions.

### Network centrality

NetworkX (https://networkx.github.io/), a network analysis framework in a Python language, was used to calculate the degrees, Estrata Index and betweenness centrality of nodes (genes/proteins).

### GBM gene expression

Gene expression data used for edge weight determination in the weighted-network were collected from five gene expression data sets for GBM patients taken from GEO (Gene Expression Omnibus)[111] GSE4290 (77 samples), GSE53733 (70 samples), GSE50161 (34 samples), GSE36245 (46 samples) and GSE15824 (12 samples) (http://www.ncbi.nlm.nih.gov/geo/). All had been acquired with the Affymetrix GPL570 platform [http://www.ncbi.nlm.nih.gov/geo/query/acc.cgi?acc=GPL570]. The raw data of the total of 239 samples were normalized across all arrays within a set using the RMA quintile normalization procedure of the Gene Pattern Expression File Creator module [112]. Averages of normalized raw expression values were used for the study.

### Construction of cross-connected network linking paths

To construct cross-connecting linking paths (CCLPs) between signaling pathway proteins (S) and metabolic pathways proteins (M), common interacting proteins from the HPPIN were used. All possible unique connections between S and M with S-M (direct S-M interaction), and S-P-M, S-P-P-M, and S-P-P-P-M where P is a common interacting protein were established for common interacting proteins up to the second level in HPPIN (Fig 3A). Detailed descriptions of all possible connecting links are provided in the Fig 3B and Table 1. Differentially expressed (i.e. up-regulated or down-regulated) proteins in EGFRvIII versus EGFRwt expressing cells obtained from proteome analyses were mapped onto the cross-connected links [Table 1]. To assess the relevance of signaling to metabolic pathway interconnections for cancer pathogenesis, connections with at least one up-regulated or down-regulated protein (U87MGvIII versus U87MG) were selected to create functional cross-connecting sub-network (CCsN) with, in all, 2,320 protein nodes and 8,914 interactions. This CCsN was used for network topology analysis.

### Calculation of signaling entropy

The local entropy of a protein in CCsN was calculated on the basis of probabilities of an interaction of that protein with its interactors determined by using the principle of mass action. The calculation of the interaction probabilities is based on the assumption that two proteins known to interact will have a higher probability of interaction when they are highly expressed. Thereby, the interaction probability (W_ij_) of two proteins in a network is proportional to the product of expression values (E of the corresponding genes (E_i_ and E_j_) [72]:

$$W_{ij}\propto E_{i}\;X\;E_{j}$$ (v)

with the expression value of CCsN genes calculated as an average expression of a gene in the data set of 239 GBM patients.

Based on *W*_*ij*_, a stochastic matrix of normalized interaction probabilities *(P*_*ij*_*)* in the network was created for signaling entropy calculation. Probability of interaction between node i and node j was calculated as

$$P_{ij}=\frac{W_{ij}}{\sum_{{k\in N}_{i}}W_{ik}}$$ (vi)

where N_i_ is interactors of node *I* with *ΣP*_*ij*_ = 1.

The local entropy of node i was calculated as

$$S_{i}=-\frac{1}{{logk}_{i}}\sum_{j\in N(i)}p_{ij}{logp}_{ij}$$ (vii)

where k_i_ is the degree of node i in the CCsN.

### Effect-on-node as CCsN weight

To incorporate the impact of the interactors of a particular protein in the cross-connected network, the node-weight of every node *i* was specified based on the categories signaling cross-talk protein (SC), rate limiting enzyme (RLE) and up- or down-regulation in U87MGvIII versus U87MG (dEXP):

$$Wi=\{1;if(dEXP=\frac{1}{3},RLE=\frac{1}{3},SC=\frac{1}{3}),\;0;else\}$$ (viii)

Effect of interactors on a node s in CCsN was defined as effect-on-node (*eff*_*s*_) depending on the node weight of the interactors up to the second level:

$${eff}_{s}=\sum_{j}^{k}\left(\sum_{i}^{n}\frac{W_{i}}{{degree}_{i}}+\frac{W_{j}}{n_{j}}\right)$$ (ix)

where *k* is the degree of node *s*, *n*_*j*_ is the degree of protein *j*, *w*_*j*_ and *w*_*i*_ are the node weights of protein *j* and *i*.

Rate-limiting enzymes (RLE) are the enzymes in metabolic pathways whose kinetics determines the overall kinetics of the entire pathway. It is usually the enzyme with the slowest enzyme kinetics. The identification of the RLE is done based on the Michaelis–Menten equation, and the established Michaelis constants (K_M_) and V_max_ of all enzymatic steps in metabolic pathways are listed in the respective biophysics and enzyme kinetic textbooks and databases.

### Calculation of CCLP scores

The CCLP for information flow from signaling pathway protein (S) to metabolic pathway protein (M) was defined to be one of the four types shown in Fig 5A, and path scores were calculated on the basis of node and edge weights of the proteins involved in a path. To select important S-M pairs, imaginary penultimate signaling and metabolic proteins are considered as starting and ending state, and path score was calculated based on a hidden Markov model (HMM) with a forward algorithm. Emission probability (*E*_*j*_), i.e. the positional probability of a protein at the particular position in that state was calculated as

$$E_{j}=\frac{S_{j}+{Eff}_{j}}{\sum_{i}^{k}\left(S_{i}+{Eff}_{i}\right)}$$ (x)

where *k* is the number of proteins in that state, *S*_*i*_ and *S*_*j*_ are normalized local entropy of proteins *i and j*, *Eff*_*i*_ is effect-on-node for protein *i*. Within the S-M pairs of a path (Fig 5B) information flow is again scored by considering all types of paths formed between single S-M pairs and calculated as

$${Path}_{score}=I\prod_{j=1}^{n}E_{j}T_{j}$$ (xi)

where *n* is number states in a path, *I* initial probability (in our case it is equal to one), *E*_*j*_ is emission probability at state j, *T*_*j*_ is transmission probability at state j (in our case it is the probability of interaction P_ij_). *Path*_*score*_ is converted into Z-score as

$$Z_{score}=\frac{X-\mu }{\sigma }$$ (xii)

where X is raw *Path*_*score*_, μ is mean, σ is the standard deviation. A cut-off of ≥1 is applied to select significant S-M pairs and their cross-connecting links.

To understand the significance of the path scores in comparison to randomly generated paths, we have performed permutations of node weights (S_ij_) and edge weights (P_ij_) and generated 20 random paths with the same number of proteins involved in the identified paths. Averages of 20 path scores are compared with the corresponding original path score. S8 Fig shows significant difference between the original and the randomly placed weighted path scores.

### *In silico* perturbation

*In silico* perturbation analysis was done by removing nodes/proteins from the network using an in-house programme for nodes/proteins present in the final weighted sub-network. All nodes in this sub-network were removed individually from the SMIN, and the impact was studied by performing the same weighted network analysis with the sub-networks generated after node removal as the starting point. Perturbation score (P_s_) was calculated in two steps. First, we calculated significant pairs using Model 1 shown in Fig 5A and significant paths using Model 2 shown in Fig 5B using SIN-N_i_ network where N_i_ is the interaction of perturbed node N. Second, path scores after perturbation (*Path*_score'_) for significant paths identified from step 1 were calculated. Perturbation score (P_s_) was defined as

$${\text{P}}_{\text{s}}=Path_{\text{score}}-Path_{\text{score}},$$

To identify nodes with significant impact after perturbation we converted perturbation scores (P_s_) into Z-score for all signaling to metabolic pathways and vice-versa. In the perturbation analysis, node and edge weights were re-calculated for networks and paths generated after removing a node (protein) from the initial interactome. New weights in Model 1 and Model 2 were used to score the new paths and to compare the scores before and after perturbation thus to identify significant nodes in the network forming connections between signaling and metabolic pathway proteins.

### Functional enrichment by over-representation analysis (ORA)

KEGG pathway based over-representation analysis (ORA) was performed using 136 up-regulated and 243 down-regulated proteins (EGFRvIII vs EGFRwt) and 45 commonly up-regulated and 25 down-regulated proteins using ‘protein coding gene set’ as the reference gene set in WebGestalt [113] web tool.

Additionally, Gene ontology (GO) based biological process and molecular function over-representation analysis was performed for the same genes. Top 20 significant (p-values <= 0.01 and <= 0.05) categories were ranked based on the false detection rate (FDR) calculated using Benjamini and Hochberg procedure.

## Supporting information

S1 Fig Degree and betweenness distribution of HPPIN and SMIN in comparison with random networks of equivalent sizes. **(A)** The degree of distribution (left) and betweenness distribution (right) of the human protein-protein interaction network (HPPIN; red color) and ten random networks (created using Erdős–Rényi model) in 10 different colors. **(B)** The degree of distribution (left) and betweenness distribution (right) of the signaling-metabolic interaction network (SMIN; red color) and ten random networks (created using Erdős–Rényi model) in 10 different colors. The number of proteins with a given degree (k) in the network (in this figure for representation, 20 is set as the upper limit for k) approximates a power-law. HPPIN follows power-law degree distribution and is a scale-free network. The betweenness centrality of a node quantifies the communication flowing through the network from node(s)/protein(s) to another using the shortest path.
(TIF)

S2 Fig Outline of label-free comparative proteomics by SDS-PAGE plus nano-HPLC ESI-Q-TOF MS/MS. (TIF)

S3 Fig Gene ontology (GO) based over-representation analysis. Panels A and B show the enriched GO biological processes, and Panels C and D the enriched molecular functions for proteins exclusively overexpressed in U87MGvIII (EGFRvIII) and U87MG (EGFRwt), respectively.
(TIF)

S4 Fig Distribution of weighted nodes and expression states of interconnected SM pairs in the step-wise filtered networks. **(A)** The number of differentially expressed proteins (dEXP), signal crosstalk proteins (SC) and rate-limiting enzymes (RLE) present in the signaling-metabolic cross-connected network, and the overlap between each group. **(B)** The numbers of signaling-metabolic (SM) pairs present in signaling-metabolic interaction network (SMIN), the GBM-specific network and the GBM-specific significant network (threshold cut off Z ≥ 1 and Z ≥ 3), and their expression states in an EGFR-mutated U87MGvIII cell line in comparison to EGFR^wt^ U87MG. The expression states are indicated as NC: no change, NA: not identified, UP: up-regulated, DOWN: down-regulated.
(TIF)

S5 Fig Overlapping distribution of SM pairs, S-M path and genes of five selected signaling-metabolic interconnection. **(A)** Distribution of pathway-specific genes of five selected signaling pathways (left) and metabolic pathways (right). **(B)** Distribution of SM pairs of the selected signaling to all metabolic pathways (left) and all signaling to five selected groups of a metabolic pathway (right). **(C)** Distribution of the genes involved in the significant signaling-metabolic cross-connected paths of five selected signaling to all metabolic pathways (left) and all signaling to five individual groups of a metabolic pathway (right).
(TIF)

S6 Fig RAS pathway proteins with metabolic pathways interconnections. RAS pathway proteins with their expression states and inter-connections with metabolic pathways via protein-protein interactors in EGFRvIII-mutated GBM mapped onto the GBM-specific significant network.
(TIF)

S7 Fig Effect of inhibitors of signaling molecule on U87MG and U87MGvIII cell migration and invasion. **(A)** Scratch wound migration assay *in vitro*. Scratch-wounds were made by a micropipette tip in a ~ 90% confluent cell and treated separately with ABL1, PLCG1, BCL2, CALM2 and CSNK IIA inhibitors with their IC50 dose (5 μM, 3 μM, 25 μM, 45 μM and 40 μM respectively) and incubated with IMDM and 1% FBS and images were taken at 0 hrs and 8 hrs. Comparisons of percent wound healing area of individual treatment group with untreated group are represented as bar diagram for respective cell line. **(B)** Matrigel coated transwell chamber migration/invasion assay *in vitro*. U87MG and U87MGvIII cells were seeded to the matrigel-coated upper invasion chamber in serum-free medium and lower chamber was filled with medium with ABL1 and PLCG1 inhibitors at their IC50 doses. The cells on the lower surface of the insert were stained with the crystal violet after 24 hrs. Comparisons of number of invaded cells between untreated and treated conditions are presented as bar diagram.
(TIF)

S8 Fig Comparison of path scores between the original and randomly assigned weighted paths. Random node weights (S_ij_) and edge weights (P_ij_) were applied to the path forming proteins to generate 20 random paths for each original path. Scores were compared between the original and the random paths for S-P-M (A), S-P-P-M (B), and S-P-P-P-M (C) connections, respectively. For panels A and B, path scores are in the range of 10^−17^.
(TIF)

S1 Table Network properties of HPPIN and SMIN with 10 random networks of equivalent size. Clustering coefficient (CC): It is a measure of the degree to which nodes in a graph tend to cluster. Assortivity: It represents to what extent nodes in a network associate with other nodes in the network, being of similar sort or of opposing sort. Estrada Index: It is a measure of the robustness of complex networks by Eigen values and Eigen vectors.
(DOCX)

S2 Table Individual signaling or metabolic pathway-wise statistics of a number of paths and pairs formed in different networks. The number of paths formed between signaling-metabolic pairs for information flow from 14 signaling pathways to all metabolic pathways (magenta) and from all signaling pathways to six groups of metabolic pathways (blue) in signaling-metabolic interaction network (SMIN) and significant GBM-specific network.
(DOCX)

S3 Table Identification of important network proteins from *in silico* perturbation studies. Number of significant (-2≥Z≥2) proteins identified from *in silico* perturbation analysis to impact information flow from 14 analyzed signaling pathways to all metabolic pathways and from all signaling pathways to 6 metabolic pathways. In X|Y, X is the number of common proteins in the indicated pathway and Y is total number of significant proteins.
(DOCX)

S1 File S-MPaths formed through signaling molecules used for inhibition study as shown in Fig 9. (XLSX)
