# Supplementary figures and images for: Connecting signaling and metabolic pathways in EGF receptor-mediated oncogenesis of glioblastoma
Source: PLoS Comput Biol. 2019 Aug 6;15(8):e1007090. doi: 10.1371/journal.pcbi.1007090 (PMC6684045; doi:10.1371/journal.pcbi.1007090)

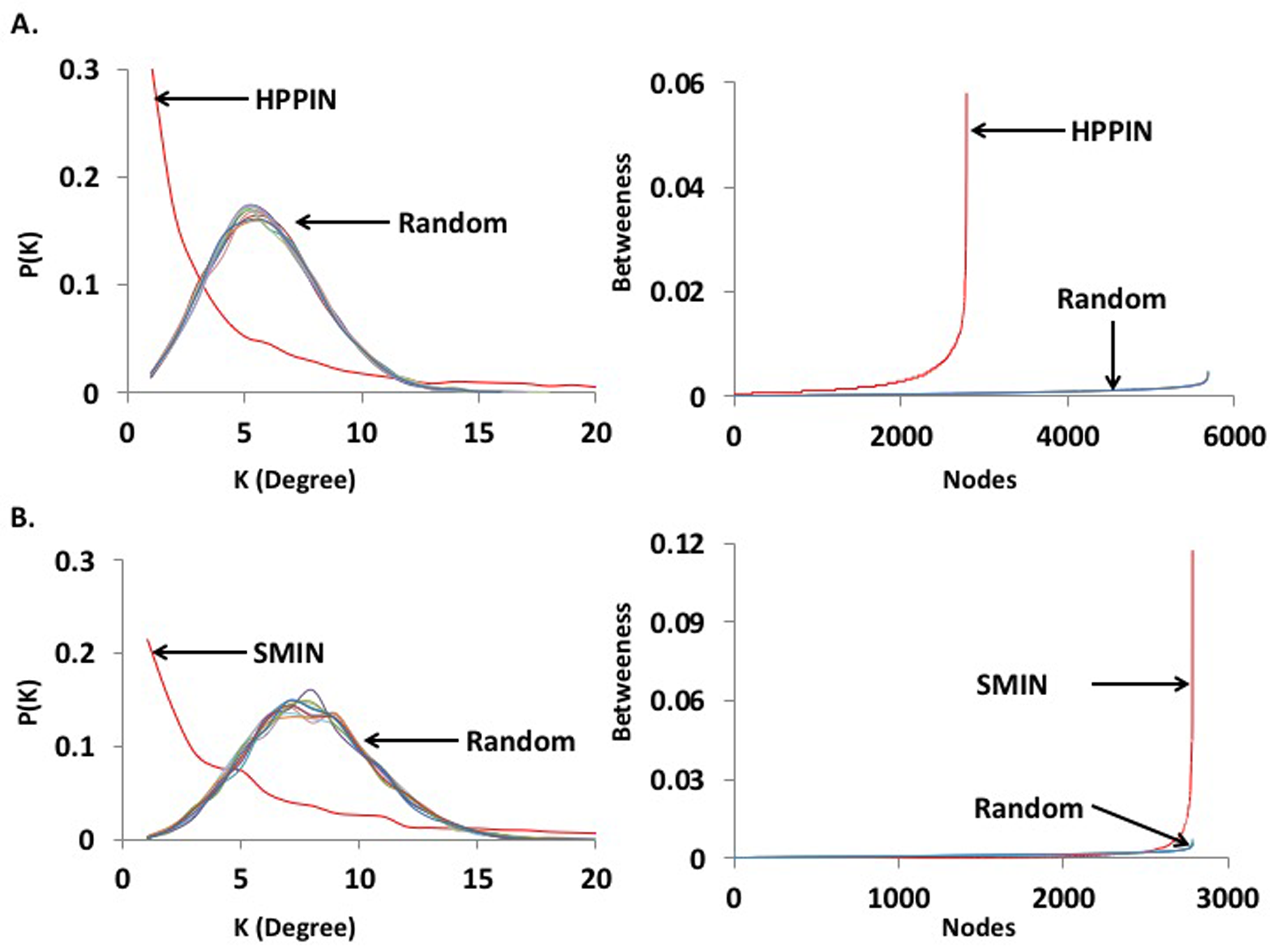

Supplement: S1 Fig — (A) The degree of distribution (left) and betweenness distribution (right) of the human protein-protein interaction network (HPPIN; red color) and ten random networks (created using Erdős–Rényi model) in 10 different colors. (B) The degree of distribution (left) and betweenness distribution (right) of the signaling-metabolic interaction network (SMIN; red color) and ten random networks (created using Erdős–Rényi model) in 10 different colors. The number of proteins with a given degree (k) in the network (in this figure for representation, 20 is set as the upper limit for k) approximates a power-law. HPPIN follows power-law degree distribution and is a scale-free network. The betweenness centrality of a node quantifies the communication flowing through the network from node(s)/protein(s) to another using the shortest path. (TIF) [file pcbi.1007090.s001.tif]

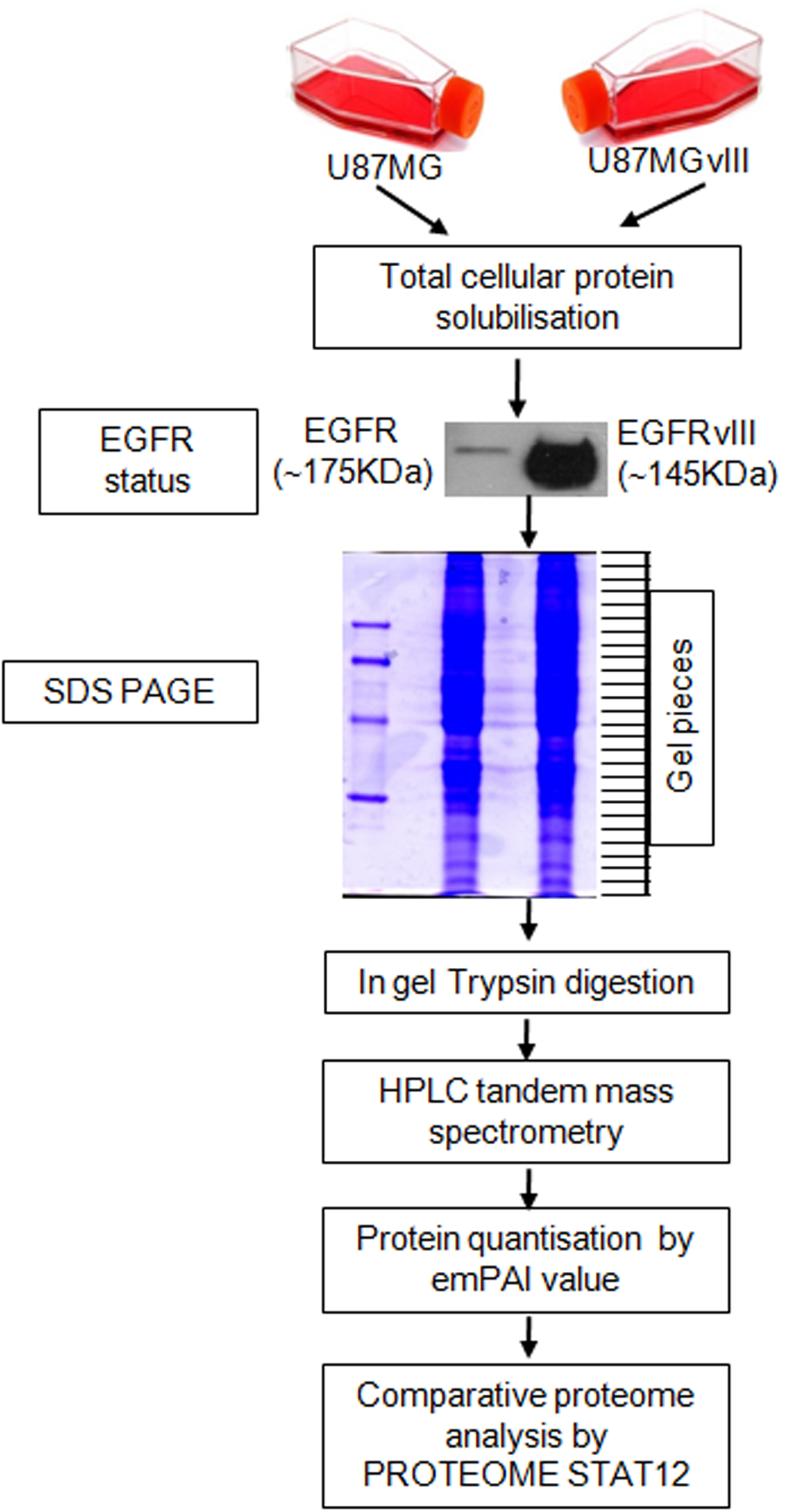

Supplement: S2 Fig — (TIF) [file pcbi.1007090.s002.tif]

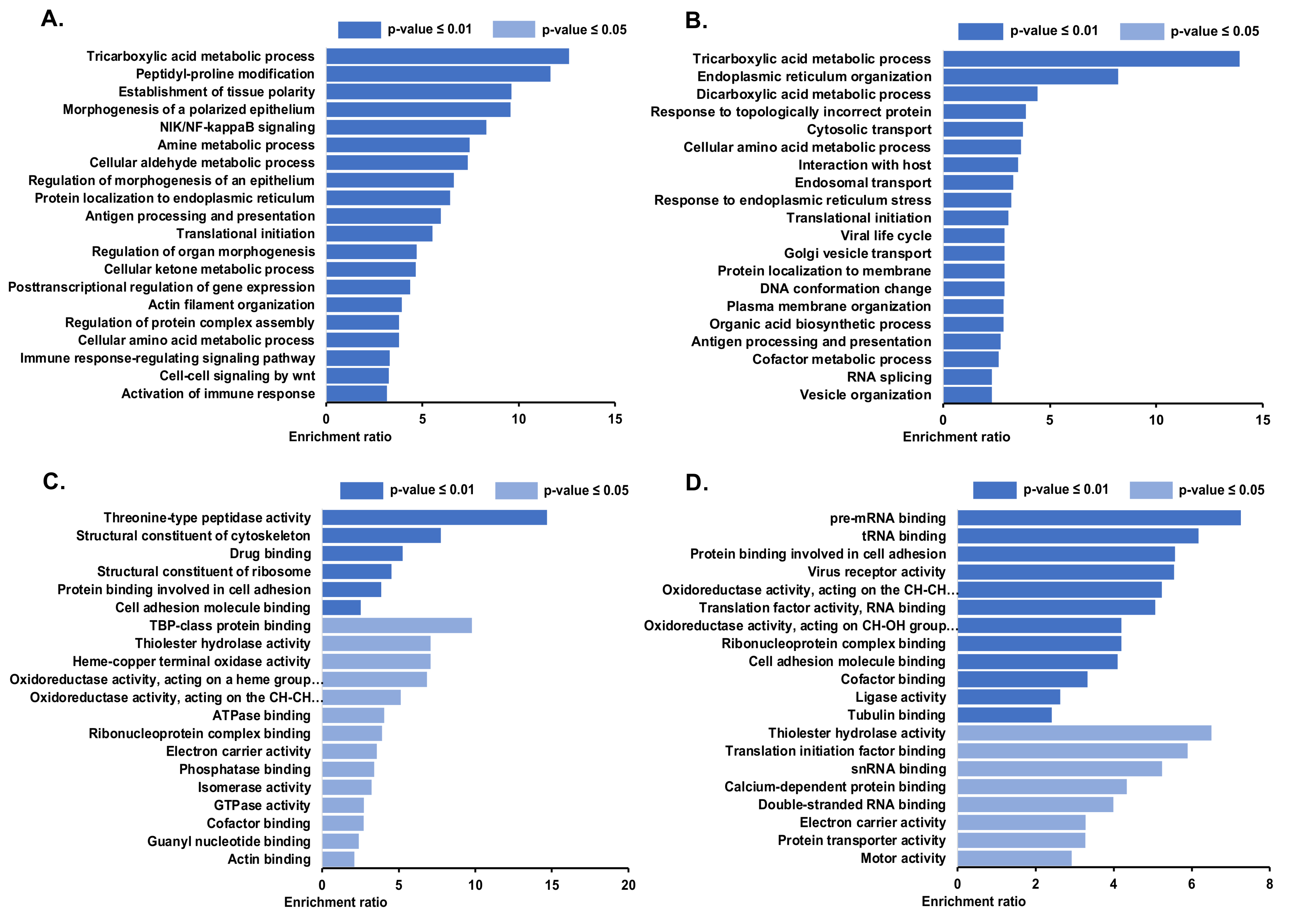

Supplement: S3 Fig — Panels A and B show the enriched GO biological processes, and Panels C and D the enriched molecular functions for proteins exclusively overexpressed in U87MGvIII (EGFRvIII) and U87MG (EGFRwt), respectively. (TIF) [file pcbi.1007090.s003.tif]

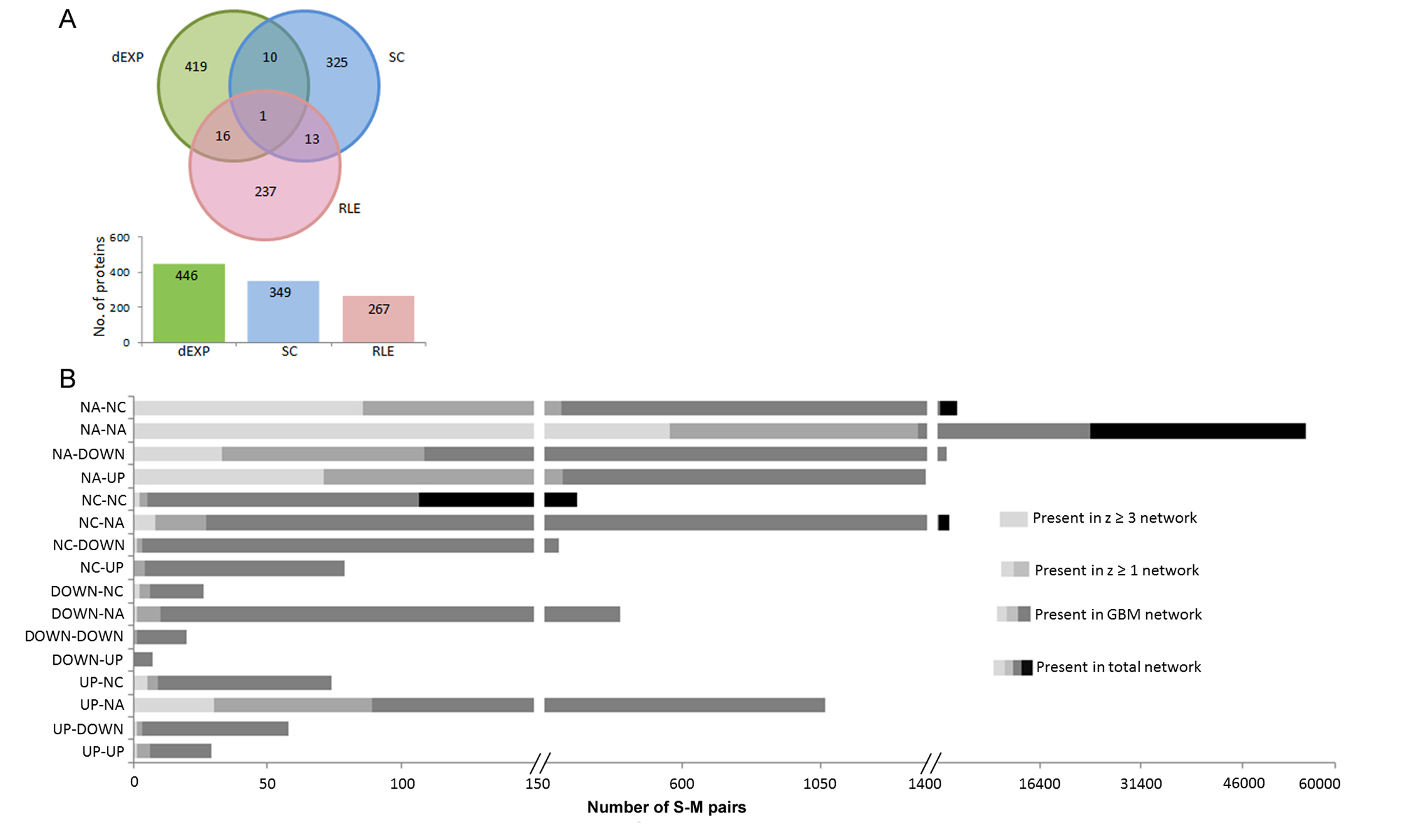

Supplement: S4 Fig — (A) The number of differentially expressed proteins (dEXP), signal crosstalk proteins (SC) and rate-limiting enzymes (RLE) present in the signaling-metabolic cross-connected network, and the overlap between each group. (B) The numbers of signaling-metabolic (SM) pairs present in signaling-metabolic interaction network (SMIN), the GBM-specific network and the GBM-specific significant network (threshold cut off Z ≥ 1 and Z ≥ 3), and their expression states in an EGFR-mutated U87MGvIII cell line in comparison to EGFRwt U87MG. The expression states are indicated as NC: no change, NA: not identified, UP: up-regulated, DOWN: down-regulated. (TIF) [file pcbi.1007090.s004.tif]

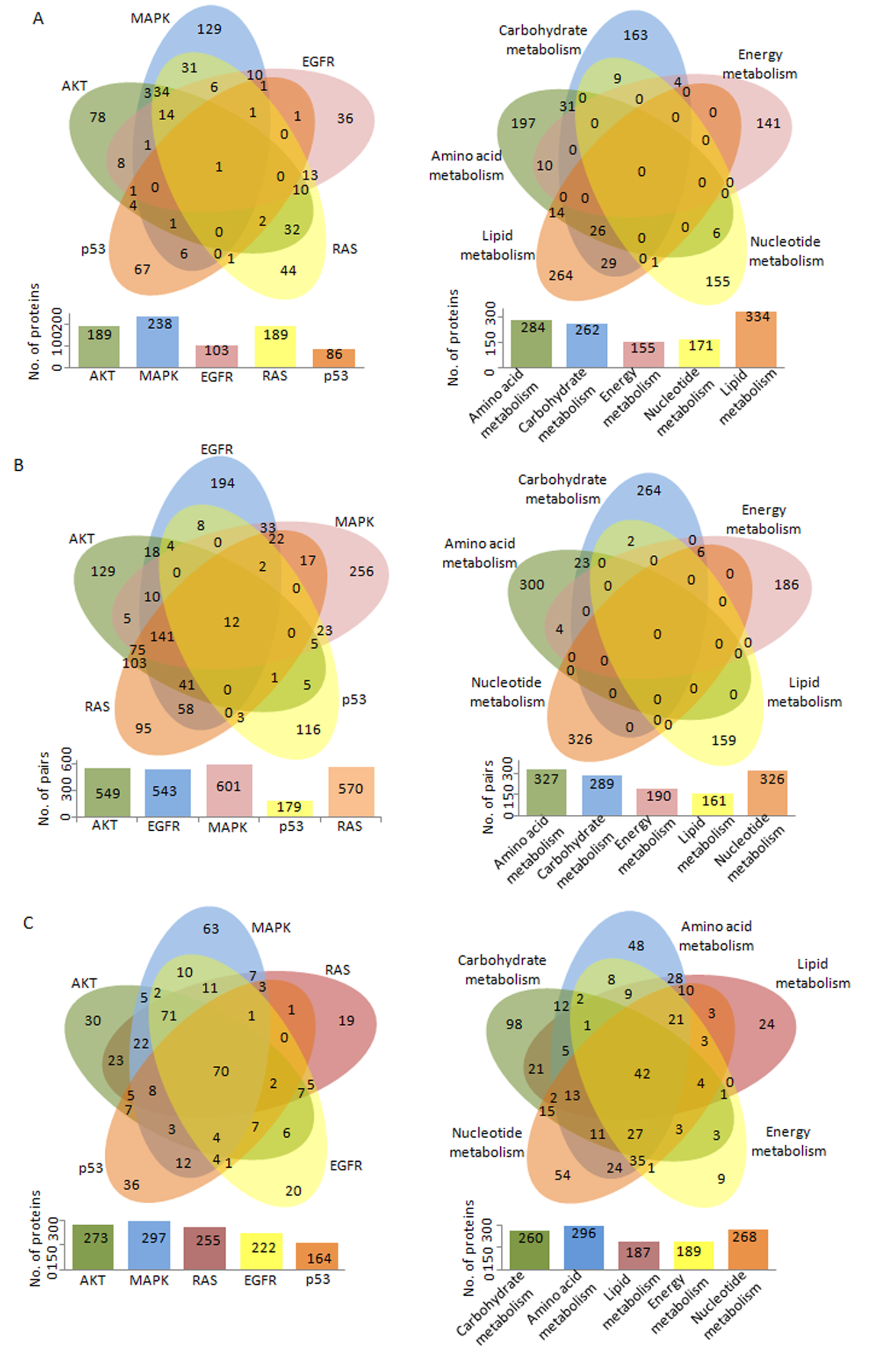

Supplement: S5 Fig — (A) Distribution of pathway-specific genes of five selected signaling pathways (left) and metabolic pathways (right). (B) Distribution of SM pairs of the selected signaling to all metabolic pathways (left) and all signaling to five selected groups of a metabolic pathway (right). (C) Distribution of the genes involved in the significant signaling-metabolic cross-connected paths of five selected signaling to all metabolic pathways (left) and all signaling to five individual groups of a metabolic pathway (right). (TIF) [file pcbi.1007090.s005.tif]

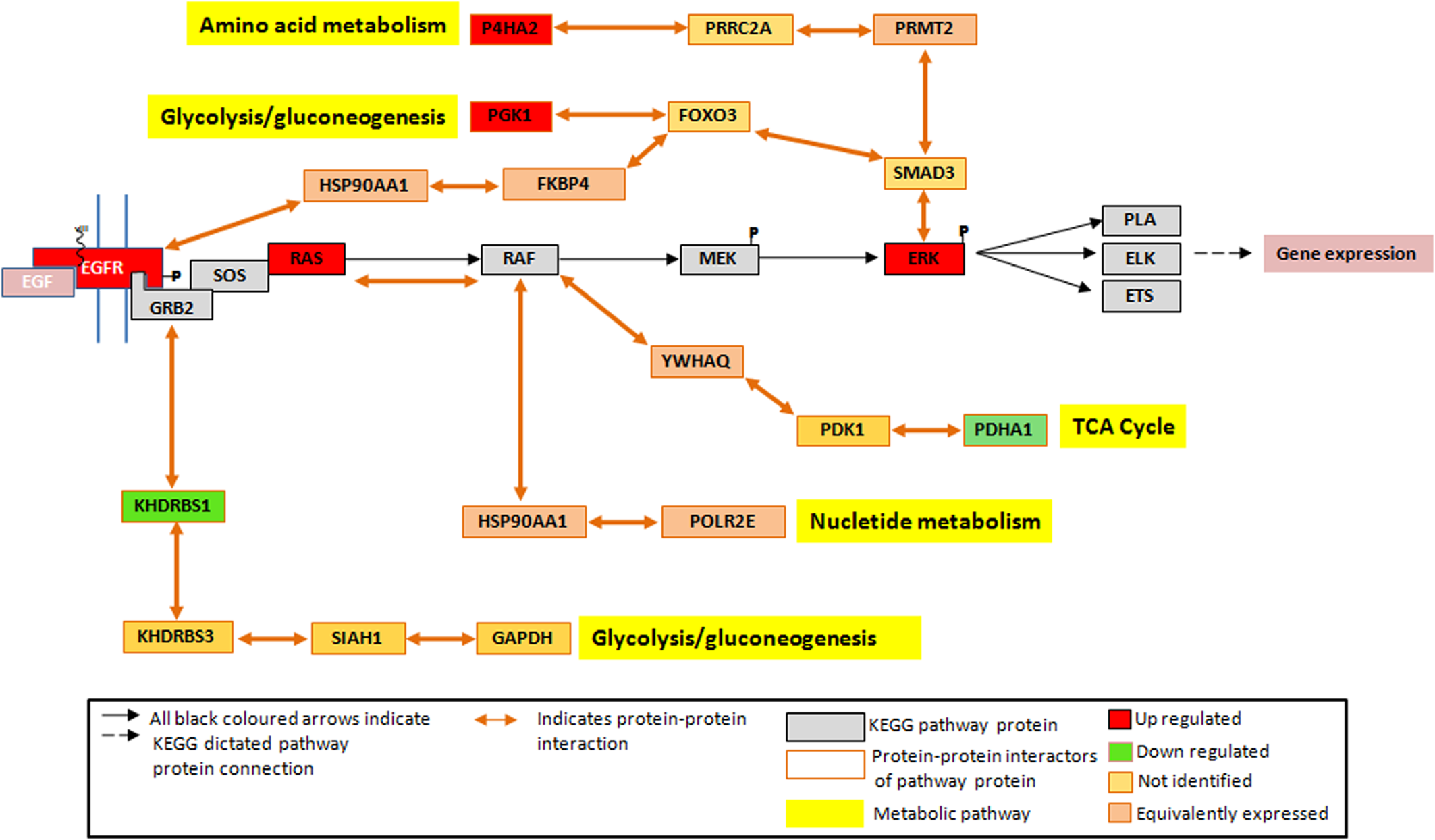

Supplement: S6 Fig — RAS pathway proteins with their expression states and inter-connections with metabolic pathways via protein-protein interactors in EGFRvIII-mutated GBM mapped onto the GBM-specific significant network. (TIF) [file pcbi.1007090.s006.tif]

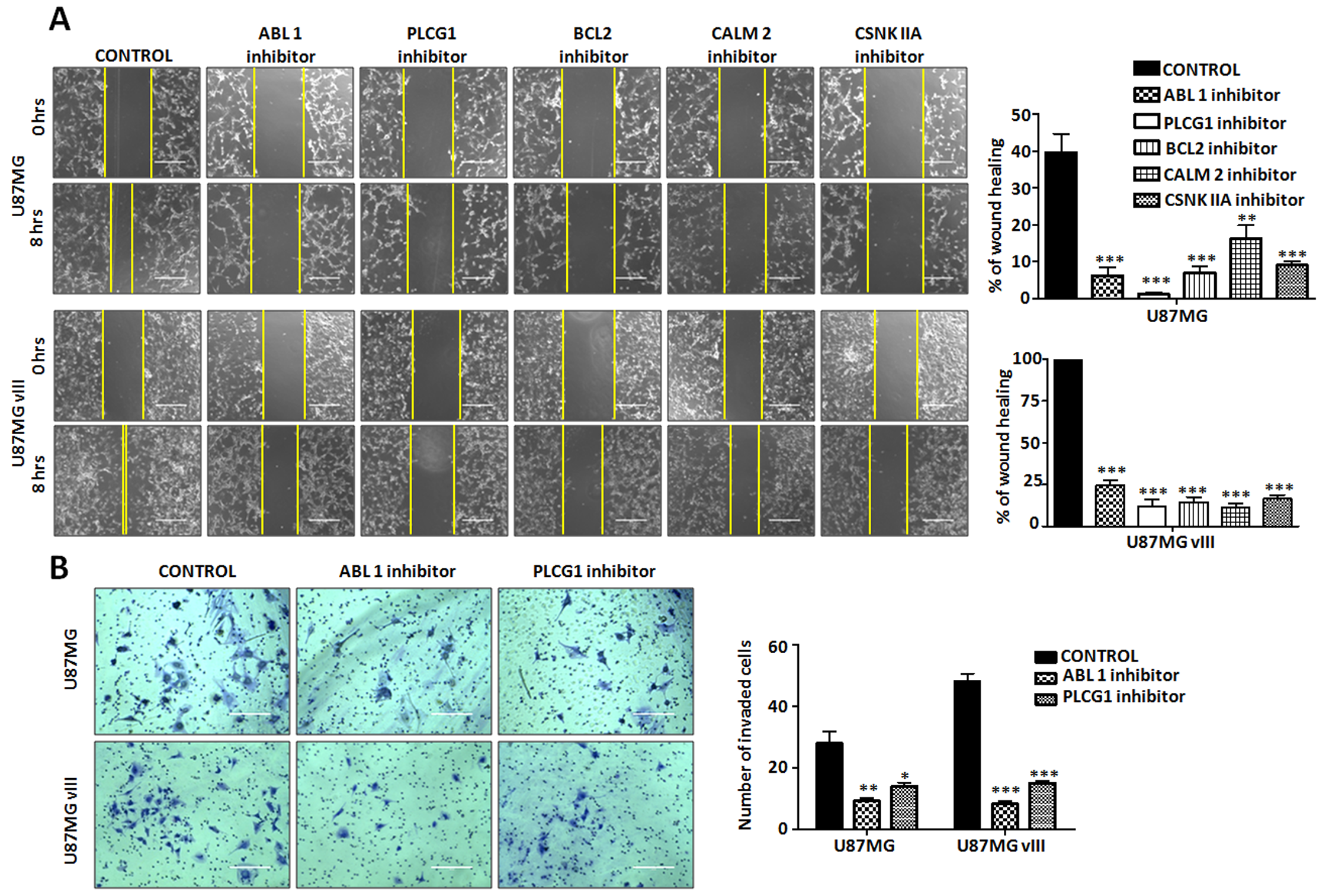

Supplement: S7 Fig — (A) Scratch wound migration assay in vitro. Scratch-wounds were made by a micropipette tip in a ~ 90% confluent cell and treated separately with ABL1, PLCG1, BCL2, CALM2 and CSNK IIA inhibitors with their IC50 dose (5 μM, 3 μM, 25 μM, 45 μM and 40 μM respectively) and incubated with IMDM and 1% FBS and images were taken at 0 hrs and 8 hrs. Comparisons of percent wound healing area of individual treatment group with untreated group are represented as bar diagram for respective cell line. (B) Matrigel coated transwell chamber migration/invasion assay in vitro. U87MG and U87MGvIII cells were seeded to the matrigel-coated upper invasion chamber in serum-free medium and lower chamber was filled with medium with ABL1 and PLCG1 inhibitors at their IC50 doses. The cells on the lower surface of the insert were stained with the crystal violet after 24 hrs. Comparisons of number of invaded cells between untreated and treated conditions are presented as bar diagram. (TIF) [file pcbi.1007090.s007.tif]

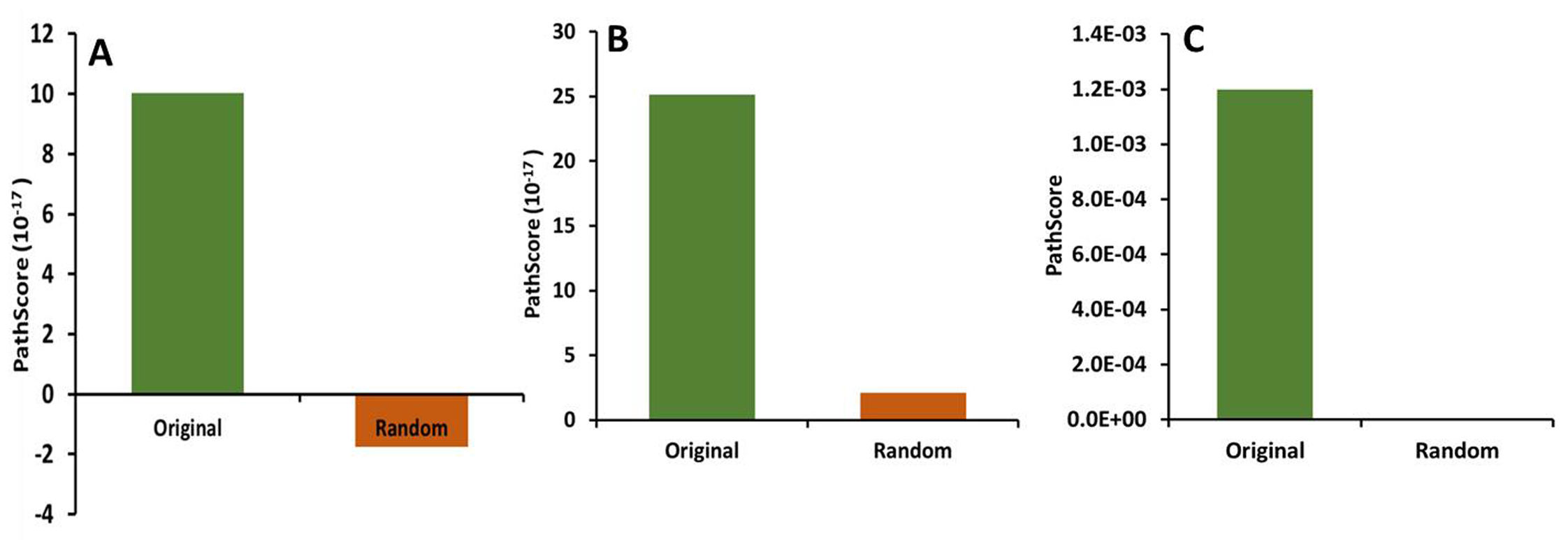

Supplement: S8 Fig — Random node weights (Sij) and edge weights (Pij) were applied to the path forming proteins to generate 20 random paths for each original path. Scores were compared between the original and the random paths for S-P-M (A), S-P-P-M (B), and S-P-P-P-M (C) connections, respectively. For panels A and B, path scores are in the range of 10−17. (TIF) [file pcbi.1007090.s008.tif]
