# Supplementary material for: Connecting signaling and metabolic pathways in EGF receptor-mediated oncogenesis of glioblastoma
Source: PLoS Comput Biol. 2019 Aug 6;15(8):e1007090. doi: 10.1371/journal.pcbi.1007090 (PMC6684045; doi:10.1371/journal.pcbi.1007090)
Supplement: S1 Table — Clustering coefficient (CC): It is a measure of the degree to which nodes in a graph tend to cluster. Assortivity: It represents to what extent nodes in a network associate with other nodes in the network, being of similar sort or of opposing sort. Estrada Index: It is a measure of the robustness of complex networks by Eigen values and Eigen vectors. (DOCX) [file pcbi.1007090.s009.docx]

**Table S1**

| **A.** | | **HPPIN**  **(16828 Edges)** | | | | |
| --- | --- | --- | --- | --- | --- | --- |
|  | | **CC** | **Assortivity** | | | **Estrada Index** |
| **Real PPI** | | 0.45 | 0.03786 | | | 1.55e^+12^ |
| **Random 1** | | 0.13 | -0.09 | | | 3.57e^-08^ |
| **Random 2** | | 0.12 | 0.10 | | | 3.59e^-08^ |
| **Random 3** | | 0.12 | -0.26 | | | 3.57e^-08^ |
| **Random 4** | | 0.13 | -0.06 | | | 3.56e^-08^ |
| **Random 5** | | 0.12 | -0.47 | | | 3.57e^-08^ |
| **Random 6** | | 0.17 | -0.18 | | | 3.58e^-08^ |
| **Random 7** | | 0.10 | 0.16 | | | 3.56e^-08^ |
| **Random 8** | | 0.13 | -0.0012 | | | 3.58e^-08^ |
| **Random 9** | | 0.11 | -0.03 | | | 3.56e^-08^ |
| **Random 10** | | 0.13 | 0.45 | | | 3.57e^-08^ |
| **B.** | **SMIN**  **(11059 Edges)** | | | | | |
|  | **CC** | | | **Assortivity** | **Estrada Index** | |
| SMIN | 0.43 | | | -0.015 | 1.40E+12 | |
| Random1 | 0.07 | | | 0.35 | 3.95E-08 | |
| Random2 | 0.08 | | | -0.02 | 3.93E-08 | |
| Random3 | 0.06 | | | 0.62 | 3.94E-08 | |
| Random4 | 0.08 | | | -0.31 | 3.98E-08 | |
| Random5 | 0.07 | | | 0.19 | 3.96E-08 | |
| Random6 | 0.07 | | | -0.80 | 3.92E-08 | |
| Random7 | 0.07 | | | 0.34 | 3.9E-08 | |
| Random8 | 0.08 | | | 0.19 | 3.99E-08 | |
| Random9 | 0.07 | | | 0.33 | 3.96E-08 | |
| Random10 | 0.07 | | | 0.24 | 3.92E-08 | |
