# Supplementary material for: Connecting signaling and metabolic pathways in EGF receptor-mediated oncogenesis of glioblastoma
Source: PLoS Comput Biol. 2019 Aug 6;15(8):e1007090. doi: 10.1371/journal.pcbi.1007090 (PMC6684045; doi:10.1371/journal.pcbi.1007090)
Supplement: S2 Table — The number of paths formed between signaling-metabolic pairs for information flow from 14 signaling pathways to all metabolic pathways (magenta) and from all signaling pathways to six groups of metabolic pathways (blue) in signaling-metabolic interaction network (SMIN) and significant GBM-specific network. (DOCX) [file pcbi.1007090.s010.docx]

Table S2

| **Pathways** | **Human interactome** | | **Network** | | | | | |
| --- | --- | --- | --- | --- | --- | --- | --- | --- |
|  |  |  | **GBM specific** | | **At low confidence (Z>=1)** | | **At high confidence k (Z>=3)** | |
|  | Number | | | | | | | |
|  | PATHS | PAIRS | PATHS | PAIRS | PATHS | PAIRS | PATHS | PAIRS |
| **Signaling Pathways** | | | | | | | | |
| **AKT** | 631,468 | 16,946 | 146,451 | 8,689 | 629 | 549 | 203 | 177 |
| **APOPTOSIS** | 468,546 | 11,900 | 69,753 | 5,558 | 323 | 298 | 108 | 97 |
| **EGFR** | 462,562 | 10,462 | 133,824 | 6,577 | 569 | 543 | 225 | 219 |
| **HH** | 43,805 | 1,768 | 19,005 | 912 | 66 | 60 | 29 | 23 |
| **JAK-STAT** | 378,673 | 8,417 | 71,213 | 4,620 | 369 | 340 | 163 | 148 |
| **JNK** | 195,035 | 4,307 | 33,477 | 2,638 | 173 | 154 | 57 | 51 |
| **MAPK** | 498,259 | 17,239 | 121,862 | 9,114 | 668 | 601 | 255 | 230 |
| **MTOR** | 128,789 | 5,401 | 37,706 | 2,844 | 152 | 135 | 52 | 50 |
| **NFKB** | 399,467 | 12,248 | 80,292 | 5,599 | 323 | 310 | 102 | 98 |
| **NOTCH** | 457,111 | 7,435 | 74,727 | 3,874 | 146 | 139 | 52 | 50 |
| **P53** | 370,654 | 8,738 | 45,991 | 4,097 | 199 | 179 | 64 | 61 |
| **RAS** | 449,497 | 12,502 | 127,408 | 6,829 | 652 | 570 | 237 | 205 |
| **TGFB** | 480,693 | 11,415 | 109,779 | 5,989 | 313 | 297 | 109 | 106 |
| **WNT** | 408,782 | 9,528 | 78,000 | 4,940 | 334 | 316 | 144 | 135 |
| **Metabolic Pathways** | | | | | | | | |
| **Carbohydrate** | 66,147 | 5,694 | 29,501 | 3,053 | 298 | 289 | 132 | 127 |
| **Lipid** | 147,267 | 7,525 | 47,749 | 4,776 | 171 | 161 | 42 | 41 |
| **Amino Acid** | 740,839 | 18,529 | 85,867 | 9,119 | 368 | 327 | 129 | 119 |
| **Nucleotide** | 490,011 | 19,610 | 62,354 | 8,545 | 344 | 326 | 125 | 120 |
| **Energy** | 44,771 | 3,655 | 5,601 | 1,113 | 199 | 190 | 78 | 75 |
| **Xenobiotics** | 20,334 | 1,810 | 2,148 | 502 | 11 | 10 | 0 | 0 |
