# Supplementary material for: Connecting signaling and metabolic pathways in EGF receptor-mediated oncogenesis of glioblastoma
Source: PLoS Comput Biol. 2019 Aug 6;15(8):e1007090. doi: 10.1371/journal.pcbi.1007090 (PMC6684045; doi:10.1371/journal.pcbi.1007090)
Supplement: S3 Table — Number of significant (-2≥Z≥2) proteins identified from in silico perturbation analysis to impact information flow from 14 analyzed signaling pathways to all metabolic pathways and from all signaling pathways to 6 metabolic pathways. In X|Y, X is the number of common proteins in the indicated pathway and Y is total number of significant proteins. (DOCX) [file pcbi.1007090.s011.docx]

**Table S3**

|  | AKT | APOP | EGFR | HH | JAK-STAT | JNK | MAPK | MTOR | NFKB | NOTCH | P53 | RAS | TGFB | WNT | AA-Met | C-Met | E-Met | L-Met | N-Met | X-Met |
| --- | --- | --- | --- | --- | --- | --- | --- | --- | --- | --- | --- | --- | --- | --- | --- | --- | --- | --- | --- | --- |
| AKT | 9\|9 | 5\|5 | 1\|10 | 1\|4 | 2\|11 | 0\|9 | 0\|8 | 6\|6 | 3\|13 | 2\|12 | 1\|3 | 1\|11 | 3\|9 | 3\|6 | 2\|3 | 5\|5 | 0\|6 | 0\|9 | 0\|2 | 0\|5 |
| APOP | 5\|9 | 5\|5 | 1\|10 | 1\|4 | 2\|11 | 0\|9 | 0\|8 | 2\|6 | 1\|13 | 0\|12 | 1\|3 | 0\|11 | 3\|9 | 3\|6 | 2\|3 | 5\|5 | 0\|6 | 0\|9 | 0\|2 | 0\|5 |
| EGFR | 1\|9 | 1\|5 | 10\|10 | 1\|4 | 4\|11 | 2\|9 | 2\|8 | 0\|6 | 0\|13 | 5\|12 | 0\|3 | 5\|11 | 2\|9 | 0\|6 | 0\|3 | 1\|5 | 5\|6 | 1\|9 | 0\|2 | 0\|5 |
| HH | 1\|9 | 1\|5 | 1\|10 | 4\|4 | 0\|11 | 0\|9 | 1\|8 | 0\|6 | 0\|13 | 0\|12 | 0\|3 | 1\|11 | 0\|9 | 0\|6 | 0\|3 | 1\|5 | 0\|6 | 1\|9 | 0\|2 | 0\|5 |
| JAK-STAT | 2\|9 | 2\|5 | 4\|10 | 0\|4 | 11\|11 | 2\|9 | 2\|8 | 2\|6 | 1\|13 | 3\|12 | 0\|3 | 2\|11 | 4\|9 | 2\|6 | 2\|3 | 2\|5 | 4\|6 | 0\|9 | 0\|2 | 0\|5 |
| JNK | 0\|9 | 0\|5 | 2\|10 | 0\|4 | 2\|11 | 9\|9 | 1\|8 | 0\|6 | 2\|13 | 2\|12 | 0\|3 | 3\|11 | 1\|9 | 0\|6 | 0\|3 | 0\|5 | 2\|6 | 1\|9 | 0\|2 | 0\|5 |
| MAPK | 0\|9 | 0\|5 | 2\|10 | 1\|4 | 2\|11 | 1\|9 | 8\|8 | 0\|6 | 3\|13 | 0\|12 | 0\|3 | 4\|11 | 3\|9 | 0\|6 | 1\|3 | 0\|5 | 1\|6 | 1\|9 | 0\|2 | 1\|5 |
| MTOR | 6\|9 | 2\|5 | 0\|10 | 0\|4 | 2\|11 | 0\|9 | 0\|8 | 6\|6 | 3\|13 | 2\|12 | 0\|3 | 1\|11 | 2\|9 | 2\|6 | 2\|3 | 2\|5 | 0\|6 | 0\|9 | 0\|2 | 0\|5 |
| NFKB | 3\|9 | 1\|5 | 0\|10 | 0\|4 | 1\|11 | 2\|9 | 3\|8 | 3\|6 | 13\|13 | 1\|12 | 0\|3 | 2\|11 | 2\|9 | 2\|6 | 1\|3 | 1\|5 | 0\|6 | 0\|9 | 0\|2 | 0\|5 |
| NOTCH | 2\|9 | 0\|5 | 5\|10 | 0\|4 | 3\|11 | 2\|9 | 0\|8 | 2\|6 | 1\|13 | 12\|12 | 0\|3 | 2\|11 | 0\|9 | 0\|6 | 0\|3 | 0\|5 | 5\|6 | 1\|9 | 0\|2 | 0\|5 |
| P53 | 1\|9 | 1\|5 | 0\|10 | 0\|4 | 0\|11 | 0\|9 | 0\|8 | 0\|6 | 0\|13 | 0\|12 | 3\|3 | 0\|11 | 1\|9 | 1\|6 | 0\|3 | 1\|5 | 0\|6 | 0\|9 | 2\|2 | 0\|5 |
| RAS | 1\|9 | 0\|5 | 5\|10 | 1\|4 | 2\|11 | 3\|9 | 4\|8 | 1\|6 | 2\|13 | 2\|12 | 0\|3 | 11\|11 | 3\|9 | 0\|6 | 0\|3 | 0\|5 | 2\|6 | 1\|9 | 0\|2 | 0\|5 |
| TGFB | 3\|9 | 3\|5 | 2\|10 | 0\|4 | 4\|11 | 1\|9 | 3\|8 | 2\|6 | 2\|13 | 0\|12 | 1\|3 | 3\|11 | 9\|9 | 4\|6 | 2\|3 | 3\|5 | 1\|6 | 0\|9 | 0\|2 | 0\|5 |
| WNT | 3\|9 | 3\|5 | 0\|10 | 0\|4 | 2\|11 | 0\|9 | 0\|8 | 2\|6 | 2\|13 | 0\|12 | 1\|3 | 0\|11 | 4\|9 | 6\|6 | 2\|3 | 3\|5 | 0\|6 | 0\|9 | 0\|2 | 0\|5 |
| AA-Met | 2\|9 | 2\|5 | 0\|10 | 0\|4 | 2\|11 | 0\|9 | 1\|8 | 2\|6 | 1\|13 | 0\|12 | 0\|3 | 0\|11 | 2\|9 | 2\|6 | 3\|3 | 2\|5 | 0\|6 | 0\|9 | 0\|2 | 1\|5 |
| C-Met | 5\|9 | 5\|5 | 1\|10 | 1\|4 | 2\|11 | 0\|9 | 0\|8 | 2\|6 | 1\|13 | 0\|12 | 1\|3 | 0\|11 | 3\|9 | 3\|6 | 2\|3 | 5\|5 | 0\|6 | 0\|9 | 0\|2 | 0\|5 |
| E-Met | 0\|9 | 0\|5 | 5\|10 | 0\|4 | 4\|11 | 2\|9 | 1\|8 | 0\|6 | 0\|13 | 5\|12 | 0\|3 | 2\|11 | 1\|9 | 0\|6 | 0\|3 | 0\|5 | 6\|6 | 1\|9 | 0\|2 | 0\|5 |
| L-Met | 0\|9 | 0\|5 | 1\|10 | 1\|4 | 0\|11 | 1\|9 | 1\|8 | 0\|6 | 0\|13 | 1\|12 | 0\|3 | 1\|11 | 0\|9 | 0\|6 | 0\|3 | 0\|5 | 1\|6 | 9\|9 | 0\|2 | 0\|5 |
| N-Met | 0\|9 | 0\|5 | 0\|10 | 0\|4 | 0\|11 | 0\|9 | 0\|8 | 0\|6 | 0\|13 | 0\|12 | 2\|3 | 0\|11 | 0\|9 | 0\|6 | 0\|3 | 0\|5 | 0\|6 | 0\|9 | 2\|2 | 0\|5 |
| X-Met | 0\|9 | 0\|5 | 0\|10 | 0\|4 | 0\|11 | 0\|9 | 1\|8 | 0\|6 | 0\|13 | 0\|12 | 0\|3 | 0\|11 | 0\|9 | 0\|6 | 1\|3 | 0\|5 | 0\|6 | 0\|9 | 0\|2 | 5\|5 |
